# Bioluminescent Properties of Semi-Synthetic Obelin and Aequorin Activated by Coelenterazine Analogues with Modifications of C-2, C-6, and C-8 Substituents

**DOI:** 10.3390/ijms21155446

**Published:** 2020-07-30

**Authors:** Elena V. Eremeeva, Tianyu Jiang, Natalia P. Malikova, Minyong Li, Eugene S. Vysotski

**Affiliations:** 1 Photobiology Laboratory, Institute of Biophysics SB RAS, Federal Research Center “Krasnoyarsk Science Center SB RAS”, Krasnoyarsk 660036, Russia; l_eremeeva@mail.ru (E.V.E.); npmal@yandex.ru (N.P.M.); 2 Key Laboratory of Chemical Biology (MOE), Department of Medicinal Chemistry, School of Pharmaceutical Sciences, Shandong University, Jinan 250012, China; tianyujiang@sdu.edu.cn; 3 State Key Laboratory of Microbial Technology, Shandong University–Helmholtz Institute of Biotechnology, Shandong University, Qingdao 266237, China

**Keywords:** photoprotein, obelin, aequorin, coelenterazine, analogues

## Abstract

Ca^2+^-regulated photoproteins responsible for bioluminescence of a variety of marine organisms are single-chain globular proteins within the inner cavity of which the oxygenated coelenterazine, 2-hydroperoxycoelenterazine, is tightly bound. Alongside with native coelenterazine, photoproteins can also use its synthetic analogues as substrates to produce flash-type bioluminescence. However, information on the effect of modifications of various groups of coelenterazine and amino acid environment of the protein active site on the bioluminescent properties of the corresponding semi-synthetic photoproteins is fragmentary and often controversial. In this paper, we investigated the specific bioluminescence activity, light emission spectra, stopped-flow kinetics and sensitivity to calcium of the semi-synthetic aequorins and obelins activated by novel coelenterazine analogues and the recently reported coelenterazine derivatives. Several semi-synthetic photoproteins activated by the studied coelenterazine analogues displayed sufficient bioluminescence activities accompanied by various changes in the spectral and kinetic properties as well as in calcium sensitivity. The poor activity of certain semi-synthetic photoproteins might be attributed to instability of some coelenterazine analogues in solution and low efficiency of 2-hydroperoxy adduct formation. In most cases, semi-synthetic obelins and aequorins displayed different properties upon being activated by the same coelenterazine analogue. The results indicated that the OH-group at the C-6 phenyl ring of coelenterazine is important for the photoprotein bioluminescence and that the hydrogen-bond network around the substituent in position 6 of the imidazopyrazinone core could be the reason of different bioluminescence activities of aequorin and obelin with certain coelenterazine analogues.

## 1. Introduction

Bioluminescence, the emission of light by the living organisms, is a phenomenon widespread in biosphere especially among marine dwellers. Generally, bioluminescence is a “product” of the chemical reaction in which the enzyme called luciferase catalyzes oxidation of the substrate, luciferin, by the molecular oxygen. There are more than 30 different bioluminescence systems known for today [1], however, roughly only one-third of them are characterized to some extent. Since bioluminescence is widely used as a powerful analytical tool in various fields of biology, medicine, and biotechnology, there is a continuing interest in searching new bioluminescent proteins as well as in detailed studying and improving the performance of the proteins for which molecular mechanism has been already ascertained.

Coelenterazine, one of the most commonly occurring and well-studied luciferins, serves as a substrate of bioluminescent reaction in many marine luminous organisms including jellyfishes, hydroids, ctenophores, copepods, soft corals, and most likely in some other sea inhabitants [1,2]. Many of these organisms may obtain coelenterazine from their diet as reported for jellyfish Aequorea [3], but perhaps, some of them synthesize the luciferin *de novo* such as the copepod Metridia [4].

Bioluminescence of a variety of marine organisms, mostly cnidarians and ctenophores, is caused by Ca^2+^-regulated photoproteins [1] that belong to the EF-hand Ca^2+^-binding protein superfamily and consist of a single polypeptide chain (~22 kDa) to which the oxygenated coelenterazine, 2-hydroperoxycoelenterazine, is tightly bound [5,6,7,8,9]. As the active photoprotein already contains the oxygenated coelenterazine, its light emission is independent of the presence of molecular oxygen in the reaction mixture, the feature that distinguishes photoproteins from classical luciferases. Furthermore, since the energy emitted as light is derived from the “charged” photoprotein, the molecule can react only once, i.e., it does not “turn over” as an enzyme does, unveiling another difference between photoproteins and luciferases. The molecular oxygen is yet involved in photoprotein bioluminescence at the step of active photoprotein formation. The active photoprotein can be produced by incubation of apoprotein and coelenterazine in the presence of O_2_ and reducing agents such as dithiothreitol or β-mercaptoethanol under Ca^2+^-free conditions [10,11]. Thus, Ca^2+^-regulated photoproteins can be regarded as monooxygenases capable of stabilizing the peroxy intermediate so efficiently that the enzyme-substrate complex in the form of an active photoprotein can be stored in the absence of calcium for up to months.

Photoprotein light-emitting reaction is greatly intensified by calcium that binding to the Ca^2+^-binding sites of a protein results in small structural changes within the active site leading to acceleration of the decarboxylation of 2-hydroperoxycoelenterazine with the elimination of CO_2_ and generation of the protein-bound product, coelenteramide, in an excited state [12]. Its relaxation to the ground state is accompanied by light emission with λ_max_ at 465–495 nm, depending on the photoprotein source. Without calcium ions all photoproteins display a very low level of light emission called the “calcium-independent luminescence” [13]. However, on calcium addition the light intensity increases up to 1 million-fold. Thus, calcium can be rather regarded as an allosteric modulator accelerating the photoprotein bioluminescent reaction than as a trigger which initiates the reaction.

Since the discovery of coelenterazine, a great number of its analogues have been synthesized in order to obtain luciferins which ensure the higher bioluminescence intensity, altered decay rates, different emission colors, etc. [1,14]. Many of these derivatives with various modifications of the imidazopyrazinone core substituents were tested as substrates of Ca^2+^-regulated photoproteins [1,14]. However, coelenterazine analogues possessing excellent bioluminescence properties and being capable of replacing a native coelenterazine turned out to be small in number. Moreover, information on the effect of modifications of various groups of coelenterazine on the bioluminescent properties of photoproteins is still fragmentary and often controversial. At the same time, clarification and classification of the effects of substrate modifications on the properties of Ca^2+^-regulated photoproteins would not only give a better understanding of the bioluminescence mechanism, but also provide a basis for targeted synthesis of novel coelenterazine analogues aiming to produce bioluminescent indicators with desired spectral and kinetic properties, as well as altered sensitivity to calcium for various applications in cell biology, experimental medicine, and biotechnology studies.

**Figure 1:**
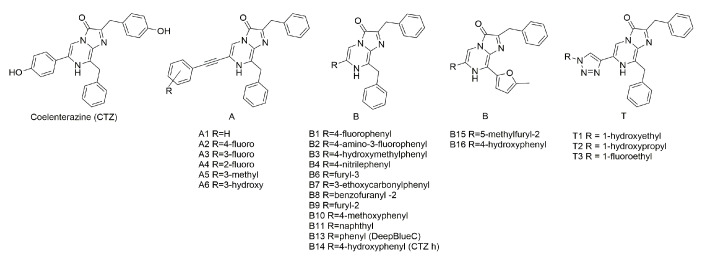
Coelenterazine analogues used in this study.

Recently, a series of novel coelenterazine analogues with different substituents at positions C-2, C-6, and C-8 of the imidazopyrazinone core have been synthesized (Figure 1) [15,16]. These compounds were tested with coelenterazine-dependent recombinant luciferases from the soft coral *Renilla reniformis* (RLuc) and the copepod *Gaussia princeps* (GLuc). Some of these analogues revealed promising properties as the substrates of these luciferases as compared to DeepBlueC™ or native coelenterazine in both *in vitro* and *in vivo* experiments.

In this paper, we report the properties (specific bioluminescent activity, light emission spectra, stopped-flow kinetics, and sensitivity to calcium) of obelin and aequorin activated by these coelenterazine analogues (Figure 1) [15,16] as well as by some novel coelenterazine derivatives (Figure 1, B15, B16).

## 2. Results and Discussion

### 2.1. Specific Bioluminescence Activity

The coelenterazine analogues (Figure 1) were tested with aequorin from *Aequorea victoria* [17,18,19] and obelin from *Obelia longissima* [20,21]. These Ca^2+^-regulated photoproteins were selected because they are the best studied photoproteins [12,22,23] and because they frequently display different properties and activities upon being activated by the same coelenterazine analogue [24]. Among the semi-synthetic photoproteins examined, only Aq_A6, Aq_B14, Aq_B15, Ol_A1, Ol_A6, Ol_B1, Ol_B3, Ol_B4, and Ol_B14 have preserved sufficient bioluminescence activities (7–80%) as compared to those of the corresponding photoproteins with native coelenterazine (CTZ) (Table 1). The activation of obelin and aequorin by other CTZ derivatives produced semi-synthetic photoproteins with very poor bioluminescence activities amounting to less than 1% of those with native CTZ.

The highest specific bioluminescence activities among the semi-synthetic photoproteins studied were obtained for those activated by analogues A6 and B14 (Figure 1); both obelin and aequorin retained 50–80% of bioluminescence activity (Table 1). Of note is that both CTZ derivatives contain OH-group at the 6-phenyl ring (Figure 1), which seems to be extremely important for the photoprotein bioluminescence. In contrast, the presence of a triple bond (A6) or the absence of the hydroxy group in the 2-benzyl ring of CTZ (B14) (Figure 1) is apparently not crucial for the bioluminescence reaction of photoproteins.

The use of CTZ analogue B15 comprising 5-methylfuryl-2 groups as C-6 and C-8 substituents of imidazopyrazinone core (Figure 1) produced semi-synthetic aequorin and obelin that retain 30.0% and 2.5% of bioluminescence activities of the corresponding photoproteins activated by native CTZ, respectively. However, with CTZ analogues B1 and B3 with 4-fluorophenyl and 4-hydroxymethylphenyl substituents at 6-position of the imidazopyrazinone core, respectively (Figure 1), the semi-synthetic obelins displayed higher activities (48.0% and 22.7% of that with native CTZ) than the corresponding semi-synthetic aequorins, whose bioluminescence activities were less than 1% compared to that of aequorin activated with native CTZ (Table 1).

Although the degree of identity between the amino acid sequences of obelin from *O. longissima* and aequorin from *A. victoria* is only 63.8% [23], the substrate-binding cavity of these photoproteins is formed by strictly conserved residues [12,22]. There are several amino acid residues that differ, but only one of them significantly affects the bioluminescent properties of obelin and aequorin. According to the photoprotein crystal structures, the 6-(p-hydroxy)-phenyl group of CTZ is in a close proximity from Phe88 in obelin and the corresponding Tyr82 in aequorin (residue is numbered according to aequorin spatial structure) [6,7].

The role of these residues was revealed by substitution of Phe to Tyr in obelin and Tyr to Phe in aequorin [25]. The F88Y mutation in obelin resulted in a shift of its bioluminescence maximum to shorter wavelengths (λ_max_ = 453 nm) and disappearance of the shoulder at 400 nm, thus making its light emission spectrum similar to that of the wild-type aequorin (λ_max_ = 465 nm). On the contrary, the Y82F aequorin displayed bioluminescence maximum at 500 nm with an additional shoulder at 400 nm, like the emission spectrum of the wild-type obelin. It was proposed that it is a hydrogen bond between OH-groups of Tyr in aequorin and the 6-(p-hydroxy)-phenyl substituent of coelenterazine that determines the differences in light emission spectra of aequorin and obelin [25]. Later, the determination of spatial structure of F88Y obelin mutant in two conformational states (before and after bioluminescent reaction) confirmed this hypothesis [26]. This hypothesis is also supported by the computational studies, which showed that the spectral properties of aequorin are significantly affected by the hydrogen bonds between His16, Tyr82, Trp86, and coelenteramide [27].

For it is quite possible that the hydrogen-bond network around the substituent in position 6 of the imidazopyrazinone core is the reason of differences in bioluminescence activities of aequorin and obelin with certain coelenterazine analogues, the bioluminescence activities of F88Y obelin and Y82F aequorin mutants activated by B1 and B15 CTZ analogues were tested. Indeed, the bioluminescence activity of F88Y obelin mutant with B15 analogue appeared to be higher when compared to wild-type obelin (Table 2). In contrast, the activation of this obelin mutant by B1 analogue caused the decreased activity. The same was observed for Y82F aequorin—the mutant activated with B1 analogue demonstrated higher bioluminescence, whereas its activity with B15 compound was reduced, i.e., aequorin mutant behaves similarly to wild-type obelin with regard to CTZ analogues (Table 2). These results clearly show that the hydrogen-bond network and other possible interactions of the residues surrounding the substituent at position 6 of the CTZ imidazopyrazinone core may influence not only the light emission color of photoproteins [25,26] but also the efficiency of bioluminescence reaction of the photoprotein in the case of its activation by CTZ analogues with modifications of this substituent.

Coelenterazine-*f* (or CTZ-*f*) is a well-known CTZ analogue with a 4-fluorobenzyl group as C-2 substituent, commonly used as a substrate because photoproteins activated by this compound display higher sensitivity to Ca^2+^ ions [28,29] and the bioluminescence activity comparable to that of photoproteins with native CTZ [29]. The B1 analogue from the present study contains 4-fluorophenyl group as the C-6 substituent, however, the bioluminescence activity of Aq_B1 is poor and Ol_B1 retains only half of the obelin activity with native CTZ (Table 1). The observed difference between CTZ-*f* and B1 analogues suggests the substituent at coelenterazine C-6 position to be of considerable importance for the photoprotein bioluminescence.

Recently, a number of novel coelenterazine analogues with various modifications of C-2, C-6, and C-8 substituents were tested with aequorin and obelin. Among those there were several analogues with 4-fluorobenzyl group as the C-2 substituent combined with another substitution at C-6 or C-8 position of coelenterazine [24]. Specific bioluminescence activities of the corresponding semi-synthetic obelins and aequorins were substantially lower compared to photoproteins activated with CTZ. There were few exceptions such as analogue with additional fluoro group on the 6-(4-hydroxyphenyl) substituent of coelenterazine displaying the same bioluminescence activity as native coelenterazine with both aequorin and obelin (97–100%) or aequorin activated with naphtyl analogue (30%) [24]. It is important to note, that in the cited study obelin and aequorin activated by the same CTZ analogue often displayed different bioluminescence activities as well.

As is mentioned above, most of the studied analogues were tested as the substrates of Renilla and Gaussia luciferases. The bioluminescence activity of RLuc with all CTZ analogues from A- and T-series was shown to be significantly lower than that with native CTZ [15]. The same was observed for most of the modified CTZs from the B-series, with the exception of B2 and B9 analogues with which RLuc exhibited 90% and 25% of native CTZ bioluminescence activity, respectively [16]. Interestingly, both aequorin and obelin showed poor bioluminescence activity with B2 and B9 analogues as compared to the Aq_WT and Ol_WT (Table 1). It is evident that while being the coelenterazine-dependent bioluminescence proteins and consequently sharing the same chemical mechanism of bioluminescence, Ca^2+^-regulated photoproteins and Renilla luciferase utilize coelenterazine and its analogues differently due to the diverse environment of the active sites and other distinguishing features of these proteins.

In addition, most of the studied coelenterazine compounds had a poor performance with GLuc, especially the analogues from A- and T-series, which displayed insignificant bioluminescence signals [15,16]. The best performance with GLuc was demonstrated by B2 analogue, however, it was only 5% of the GLuc bioluminescence with native CTZ. The strikingly different performance of the CTZ analogues with two coelenterazine-dependent luciferases, RLuc and GLuc, once again indicates the great influence that protein microenvironment has on the interaction with luciferin.

### 2.2. Bioluminescence and Fluorescence Spectra

**Figure 2:**
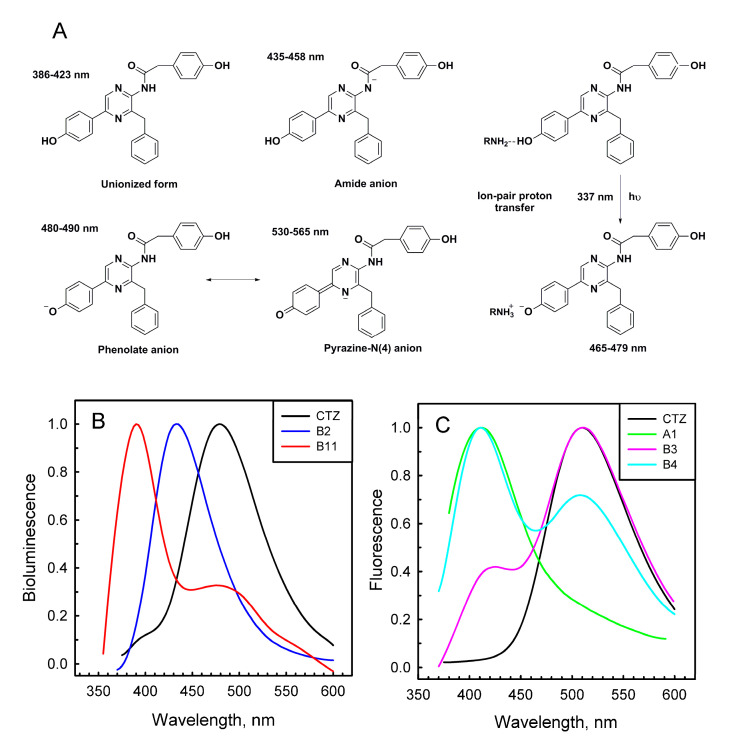
Fluorescent emitters of coelenteramide [30] (**A**), bioluminescence (**B**), and fluorescence (**C**) spectra of obelins activated with native coelenterazine and some coelenterazine analogues.

Most of the semi-synthetic aequorins displayed bioluminescence spectra similar to that of Aq_WT with a maximum around 460–470 nm (Table 1). There were only two exceptions—Aq_A2 and Aq_B2, which revealed light emission maxima shifted to shorter wavelengths (λ_max_ at 446 and 452 nm, respectively). It should, however, be mentioned that the bioluminescent activities of these semi-synthetic aequorins were very low (Table 1). In contrast, when CTZ analogues were used as obelin substrates, bioluminescence spectra of the corresponding semi-synthetic photoproteins were significantly influenced. The emission at 390 nm corresponding to that from the neutral coelenteramide [1], clearly visible in the bioluminescence spectrum of Ol_WT, disappeared in the spectra of Ol_A2-A5, Ol_B2, Ol_B14, and Ol_B16 (Table 1, Figure 2B). At the same time bioluminescence maxima of Ol_A2-A5 and Ol_B2 were shifted toward shorter wavelengths with λ_max_ around 430–445 nm. The most significant changes in the light emission spectrum were detected for obelin activated with B11 analogue, which has a naphthyl substituent in the C-6 position of imidazopyrazinone core. Ol_B11 emitted light with λ_max_ at 390 nm and a shoulder at 482 nm (Figure 2B), i.e., in fact, its bioluminescence spectrum was the “inverse copy” of that of Ol_WT. Light emission spectra of other semi-synthetic obelins were very similar to that of Ol_WT (Table 1). Of note is that semi-synthetic photoproteins with the highest bioluminescence activities did not show any promising spectral shifts (Table 1, Figure S1). According to a recent study performed on semi-synthetic aequorins and obelins, only the analogues containing electron-donating groups (m-OCH3 and m-OH) on the C-6 phenol moiety or an extended resonance system at the C-8 position (1-naphthyl and α-styryl analogues) provided a significant red shift of light emission, up to 44 nm in the case of obelin and α-styryl analogue [24].

The Ca^2+^-regulated photoproteins with peroxy adducts in the active sites are non-fluorescent, but after the bioluminescence reaction ceases they become able to fluoresce at visible wavelengths for as long as the reaction product, coelenteramide, remains bound within a substrate-binding cavity of the photoprotein. Aequorin and obelin variants displayed various types of fluorescence spectra, with maxima ranging from 405 nm to 510 nm, both monomodal and bimodal with different ratios suggesting the presence of different coelenteramide forms (Table 1, Figure 2C and Figure S2).

It was proposed that coelenteramide, the product of the bioluminescence reaction, can exist in different ionic forms [30]. These are a neutral species with a fluorescence emission maximum around 400 nm, an amide anion with a maximum around 450 nm, a phenolate anion with a maximum around 480–490 nm, and a pyrazine-N(4) anion resonance form with a maximum in the 535–550 nm range (Figure 2A). In the photoprotein bioluminescence reaction, neutral coelenteramide is believed to be the primary excited product, while the light at longer wavelengths (λ_max_ = 460–495 nm) originates from the excited phenolate anion arising from the proton dissociation from the hydroxyl group of the 6-(p-hydroxy)-phenyl substituent of coelenteramide in the direction to His22, which is located nearby at a hydrogen-bond distance [12,26]. The hybrid quantum mechanics and molecular mechanics methods combined with the molecular dynamics method confirmed experimental conclusions that the neutral form of coelenteramide is the primary excited state product and that the excited phenolate anion coelenteramide is the main aequorin emitter [31,32].

It is well known that the pK* of a phenolic group is several units below its p*K* in the ground state [33]. If this pK* falls well below 6.5, which is the expected p*K* of His, rapid transient proton dissociation and its “transient displacement” toward the N atom of His will occur, with simultaneous generation of the excited phenolate anion. Since its fluorescence lifetime is 5–6 ns [34], there is more than enough time for the proton to dissociate before radiation. However, in addition to the His residue, the OH-group of the phenol attached at C-6 of coelenterazine is also surrounded by Trp and Tyr in aequorin or Trp and Phe in obelin within hydrogen-bonding distance which also affect the spectral properties of photoproteins. It is believed that a reason for the lack of 400-nm shoulder in the aequorin bioluminescence spectrum is a more effective proton dissociation due to the additional H-bond between the OH-group of Tyr82 (Phe in this position in obelin) and *p*-OH of the phenol attached at C-6 of the 2-hydroperoxy adduct of coelenterazine.

Thus, the variety of fluorescence spectra of the Ca^2+^-discharged semi-synthetic photoproteins could be a result of improper orientation of the corresponding peroxy adducts in the active site that might disturb the environment of the substituent at C-6 position or increase the distance between this substituent and His22, thus tampering the proton dissociation. It is also interesting to note that most of the studied coelenterazine analogues, especially those of A- and T-series, tend to produce a neutral coelenteramide as a fluorescence emitter with a maximum around 400–420 nm despite the fact that in most cases the main bioluminescence emitter was detected to be a phenolate anion with a maximum around 470–480 nm (Table 1).

### 2.3. Absorbance Spectra of Coelenterazine Analogues in Ethanol, and Active and Ca^2+^-Discharged Semi-Synthetic Photoproteins

To address the matter of whether semi-synthetic photoproteins with low bioluminescence activity can even form an active photoprotein complex we determined the absorbance spectra of CTZ analogues in ethanol, as well as active and Ca^2+^-discharged photoproteins.

Native coelenterazine in ethanol (or methanol) has the absorption spectrum with a maximum at 434 nm with a small shoulder at 350 nm (Table 3, Figure S3). Photoproteins activated by CTZ display red-shifted broad absorption maximum with λ_max_ around 460 nm conditioned by 2-hydroperoxy adduct of CTZ bound within the active site. After bioluminescence reaction ceases, the absorption at 460 nm disappears and the maximum at 335 nm corresponding to the bound reaction product, coelenteramide, becomes apparent (Figure S3).

Similar to native CTZ, all the analogues in ethanol have absorbance spectra with maxima/shoulders in the visible and near-UV regions (Table 3). However, for more than half of them the absorption at near-UV region was higher than that in the visible region. Since the higher absorption in the near-UV region was probably conditioned by the product arising as a result of spontaneous oxidative decarboxylation of CTZ analogues, we can reasonably assume that many of these analogues were unstable in solution in the presence of oxygen and consequently this could be one of the reasons accounting for a very low activity of the corresponding semi-synthetic photoproteins. The other reasons could be an improper spatial orientation of the peroxy adduct in the coelenterazine-binding site or the disruption of the interactions between the key amino acid residues and analogues of coelenterazine due to the chemical modifications of the latter.

The absorbance spectra of active semi-synthetic photoproteins are diverse and depend on CTZ analogue used for activation (Table 3). For example, both Ol_A6 and Aq_A6 display red-shifted absorbance maxima at 460 nm, which match exactly those of Ol_WT and Aq_WT. A similar red shift of the absorbance maxima was observed for Ol_A1 and Ol_B1. However, the absorbance spectrum of Ol_B3 was shifted to a shorter wavelength (λ_max_ = 420 nm) as compared to that of B3 analogue in ethanol (Table 3). Both Ol_B14 and Aq_B14 revealed the same absorbance spectra as the B14 analogue has in ethanol with one distinction—whereas semi-synthetic photoproteins had a higher absorption in the near-UV region with a shoulder at 434 nm, analogues in ethanol displayed the absorbance spectrum with λ_max_ at 434 nm and a shoulder at 344 nm (Table 3). Also noteworthy is that a similar case is observed for ctenophore photoproteins, for which absorption spectrum corresponds to that of native CTZ in methanol [35]. The most unusual absorption spectrum appeared to be shown by aequorin with analogue B15 (Figure 1) comprising the two methylfuryl groups with the absorption maximum at 400 nm (Table 3). Of note is that the absorbance spectra of the above-mentioned semi-synthetic photoproteins were undoubtedly conditioned by the bound 2-hydroperoxy adduct of the corresponding CTZ analogue since all of them retained 10–80% of bioluminescence activity compared to photoproteins activated by native CTZ (Table 1).

The absorbance spectra of semi-synthetic photoproteins with low bioluminescence activities (Table 1) can be divided into two groups. Both aequorin and obelin with A2-A5, B4, B6, B8-B10, B16, or T1-T3 analogues displayed spectra matching those of the Ca^2+^-discharged photoproteins, i.e., bound with the reaction product (Table 3). It implies that many of these analogues could be unstable in solution or fail to form a stable peroxy adduct within the substrate-binding cavity of the photoprotein. Another group includes the analogues that produce semi-synthetic photoproteins with different absorption spectra for the active and discharged forms in the case of aequorin or obelin. For example, after the bioluminescence reaction the absorption spectrum of Aq_B7 significantly changed, while Ol_B7 displayed the absorption spectrum matching that of its Ca^2+^-discharged variant (Table 3). The same pattern was observed for B11 and B13 analogues. It is possible that in the absorption spectra of these semi-synthetic photoproteins the band specific for a bound 2-hydroperoxy adduct of coelenterazine analogue is located at different wavelengths as compared to the photoproteins with native coelenterazine. The differences in the absorption spectra of obelin and aequorin with these analogues may be due to the hydrogen-bond network and the residues surrounding the substituent at position 6 of the CTZ imidazopyrazinone core of a substrate in these photoproteins, i.e., similar to what was demonstrated for B1 and B15 analogues (Table 2).

Thus, the low activity of certain semi-synthetic obelins and aequorins can be brought about by several key reasons, among which are instability of some analogues in solution and environment of substituent at C-6 position of the CTZ imidazopyrazinone core influencing the efficiency of 2-hydroperoxy adduct formation.

### 2.4. Sensitivity of Semi-Synthetic Obelins and Aequorins to Calcium

The main application of Ca^2+^-regulated photoproteins is based on their ability to emit light on Ca^2+^ binding. Five decades ago, aequorin was used as an intracellular Ca^2+^ indicator for the first time and since then Ca^2+^-regulated photoproteins have been widely applied to detect calcium ions in biological systems [36]. Successful cloning of the corresponding cDNAs allowed intracellular expression of the recombinant apophotoproteins. After external addition, coelenterazine diffuses into the cells and forms an active photoprotein. Such cells with a “built-in” calcium indicator can be used to measure intracellular Ca^2+^ concentration with high efficiency, and this approach has many advantages over fluorescent Ca^2+^ probes [37].

**Figure 3:**
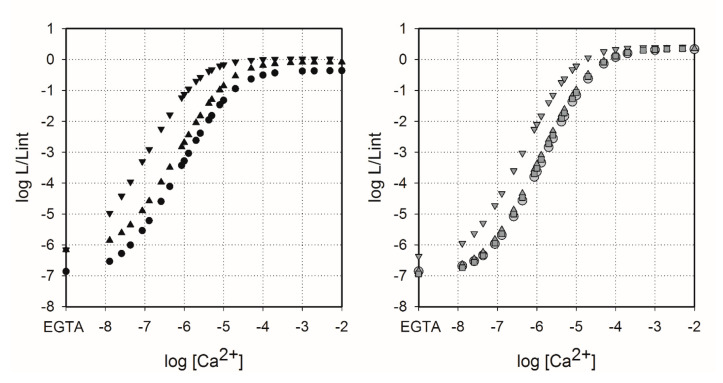
Ca^2+^ concentration-effect curves for semi-synthetic aequorins (left panel, black) and obelins (right panel, gray) activated with native CTZ (circles) and CTZ analogues A6 (triangles up), B14 (triangles down), and B1 (squares). L, light intensity at the particular Ca^2+^ concentration; L_int_, total light intensity at saturating Ca^2+^ concentration.

It is well known that the use of CTZ analogues instead of native CTZ might change sensitivity of photoproteins to Ca^2+^ [24,38]. Hence, we determined calcium sensitivity of Aq_A6, Aq_B14, Ol_A6, Ol_B1, and Ol_B14 (Figure 3) since only these semi-synthetic photoproteins have shown bioluminescence activities high enough for reliable measurements of light signals over the explored [Ca^2+^] range (Table 1). The vertical ranges of the Ca^2+^ concentration-effect curves for semi-synthetic obelins and aequorins were very similar to those of photoproteins activated by native CTZ (the vertical range of the curves spans approximately 7 log units) with small distinctions—the vertical ranges of Ol_B14 and Aq_B14 as well as of Aq_A6 were slightly reduced for 0.3 and 0.45 log units, respectively, on account of the increase of Ca^2+^-independent bioluminescence (EGTA condition, left part of the curves). Of note is that all the Ca^2+^ concentration-effect curves had the same maximum slope of approximately 2.5, meaning that three calcium ions are needed to bind with photoprotein to initiate its light emission [13,39].

The Ca^2+^ concentration-effect curves for Ol_WT, Ol_A6, and Ol_B1 were almost superimposed (Figure 3). Only for Ol_B14, the curve showed a clear difference, since it was shifted to the region of lower Ca^2+^ concentration. The same effect was observed for both semi-synthetic aequorins, however, the curve for Aq_A6 was shifted to the region of lower Ca^2+^ concentration more than the corresponding curve for Ol_A6 (Figure 3). Thus, among the semi-synthetic photoproteins tested only Aq_B14 and Ol_B14 clearly displayed higher Ca^2+^ sensitivity compared to that of the corresponding photoproteins activated by native CTZ. It is worth noting that while bioluminescence activities of semi-synthetic obelins with CTZ-*f* and B1 analogues were different, the effect of these substrate modifications on calcium sensitivity was quite similar [29].

**Figure A1:**
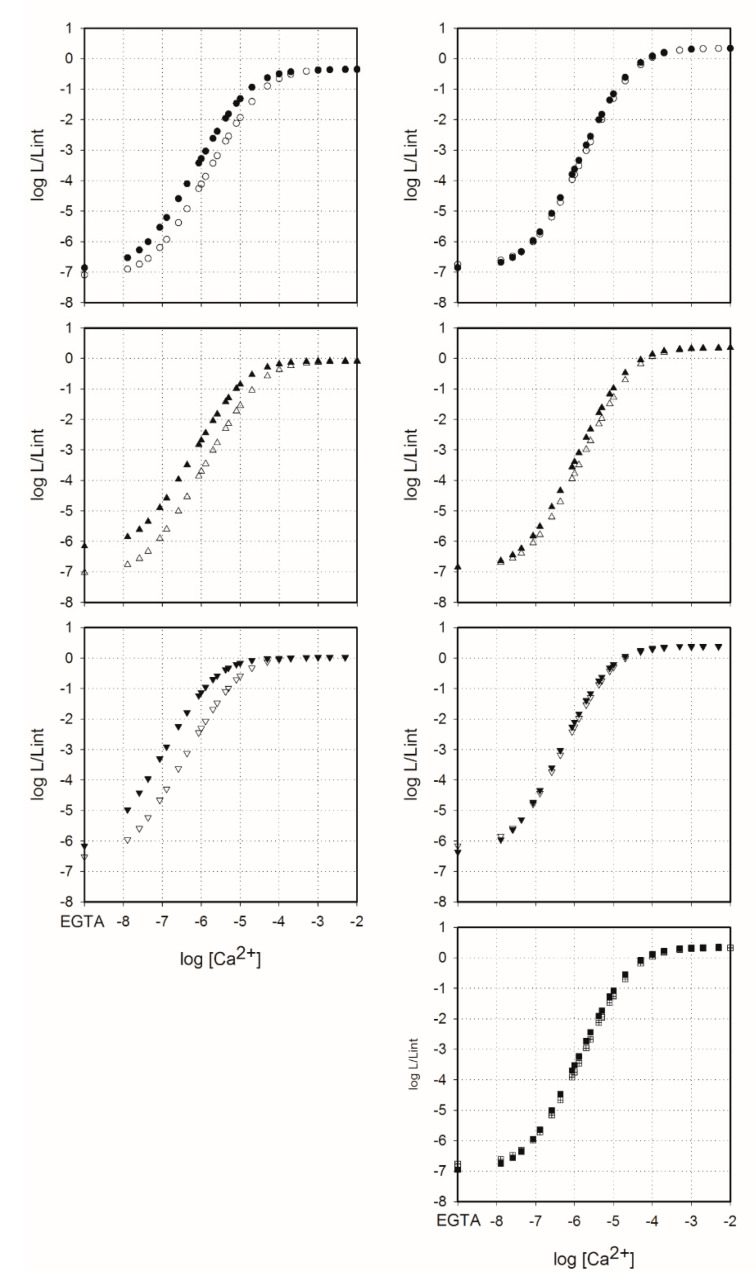
Ca^2+^ concentration–effect curves for semi-synthetic aequorins (left panel) and obelins (right panel) activated with native CTZ (circles), A6 (triangles up), B14 (triangles down), and B1 coelenterazine analogues (squares) with (*open*) and without (*filled*) 1 mM Mg^2+^. All photoproteins were pre-equilibrated with 1 mM of Mg^2+^ for 1 h before measurements. *L* and *L_int_* are the peak bioluminescence intensity for a given [Ca^2+^] and the total bioluminescence recorded for a saturating Ca^2+^ dose for the same sample, respectively.

In cells, the concentration of free Mg^2+^ exceeds that of Ca^2+^ by several orders of magnitude [40] and, being a competing ion, Mg^2+^ may decrease Ca^2+^ affinity of the Ca^2+^-binding sites of many EF-hand Ca^2+^-binding proteins. Therefore, the effect of physiological concentration of Mg^2+^ ions on the Ca^2+^ affinity of semi-synthetic photoproteins was studied (Table 4). Mg^2+^ ions noticeably affected only aequorin and its variants; the presence of magnesium ions shifts the Ca^2+^ concentration-effect curves to the right relative to those without Mg^2+^. Moreover, Mg^2+^ ions also decreased Ca^2+^-independent luminescence of aequorins (Figure A1) and increased the [Ca^2+^]_limit_ and *K*_d_ approximately twofold, even more in the case of Aq_B14 (Table 4). The effect of 1 mM Mg^2+^ on the Ca^2+^ concentration-effect relations of semi-synthetic obelins was expectedly less pronounced [24,41].

### 2.5. Rapid-Mixing Kinetics

The speed with which the luminescence responds to rapid changes in Ca^2+^ concentration is an important characteristic of Ca^2+^-regulated photoproteins. It defines the ability of photoproteins to properly track the intracellular calcium transients sometimes occurring within the millisecond timescale [42,43]. Only a few semi-synthetic photoproteins possessing bioluminescence activities high enough for stopped-flow measurements were tested (Table 5). When A6, B1, or B14 analogue was used as a substrate of obelin, the rate constants characterizing rise and decay of the light signal were not significantly influenced. In contrast, bioluminescence kinetics of Aq_A6 strikingly differed from that of Aq_WT (Table 5). The rise constant of this semi-synthetic aequorin turned out to be 2.5 times faster than that of the aequorin with native CTZ. It is interesting to note that a similar 2.3-fold increase of rise constant was found earlier for the aequorin activated by CTZ-*i* analogue [29]. In addition, the decay kinetics of this aequorin variant was satisfactorily described by a two-exponential decay function and consequently by two rate constants—“fast” (*k*_1_) and “slow” (*k*_2_), i.e., similar to all studied obelins (Table 5). According to the recently proposed unanimous kinetic model for photoprotein bioluminescence, which incorporates the “positive cooperativity” between the Ca^2+^-binding sites II and III [44], the fast decay component of bioluminescence was attributed to the intermediate that arises after calcium binding with the unpaired N-terminal Ca^2+^-binding site I. Thus, appearance of the “fast” component in the decay kinetics of light signal of Aq_A6 might be attributed to significant structural changes in the N-terminal domain, owing to the coelenterazine analogue used as a substrate.

## 3. Materials and Methods

### 3.1. Materials

Native coelenterazine was obtained from NanoLight Technology, a division of Prolume Ltd. (Pinetop, AZ, USA). Other chemicals, unless otherwise stated, were from Sigma–Aldrich and were the purest grade available.

### 3.2. Synthesis of Coelenterazine Analogues

All the reactions were monitored by TLC with 0.25 mm silica gel plates (60GF-254). UV light, iodine stain, and ninhydrin were used to visualize the spots. Silica gel was utilized for column chromatography purification. ^1^H NMR and ^13^C-NMR were recorded on a Bruker DRX spectrometer at 300 or 400 MHz, δ in parts per million, and J in hertz, using TMS as an internal standard. ESI-MS spectra were recorded on an API 4000 spectrometer. Melting points were determined uncorrected on an electrothermal melting point apparatus. HPLC tests were measured with Agilent Technologies 1260 liquid chromatography.

**Figure 4:**
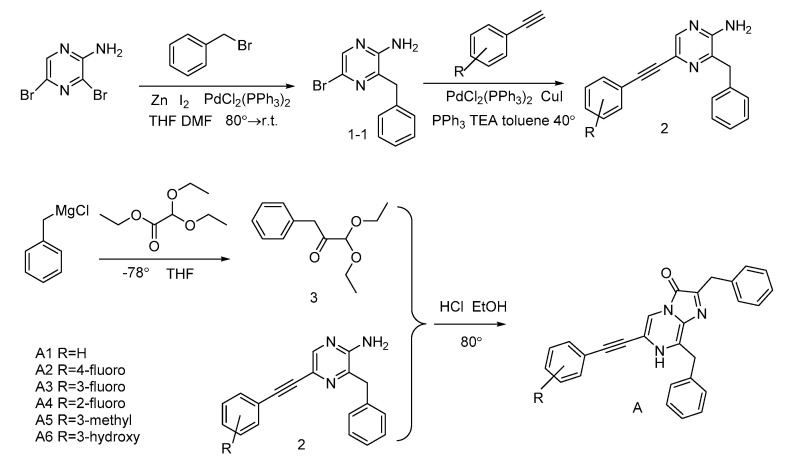
Synthesis of the A-series CTZ analogues.

For most of CTZ analogues, substituents on the C-6 phenyl ring of coelenterazine were introduced by first preparing 2-amino-3-benzyl-5-bromopyrzine (**1-1**) via regioselective palladium-catalyzed Neigishi-type cross-coupling of benzylzinc bromide and 2-amino-3,5-dibromopyrazine. For the A-series CTZ analogues [15] (Figure 4), key precursors **2** were obtained with a good yield via Sonogashira coupling reactions with substituted phenylacetylene.

**Figure 5:**
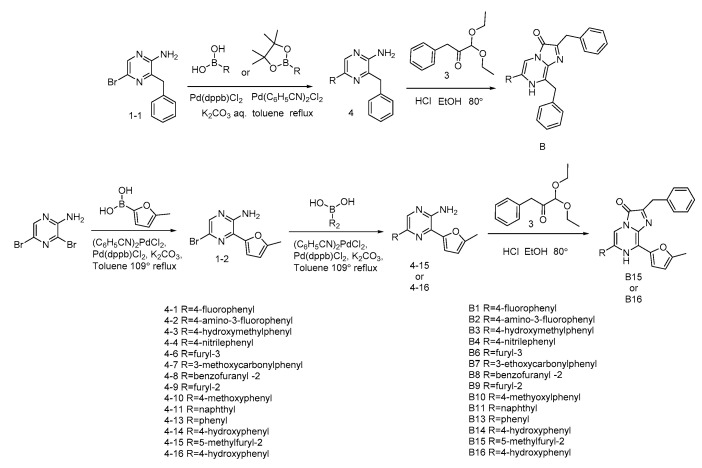
Synthesis of the B-series CTZ analogues.

For the B-series CTZ analogues [16] (Figure 5), the key intermediates **4** were synthesized with good to moderate yields by Suzuki coupling reaction with the appropriately substituted arylboronic acid. However, compounds **4-15** and **4-16** were generated from compound **1-1** and substituted arylboronic acid using two sequential Suzuki coupling reactions.

**Figure 6:**
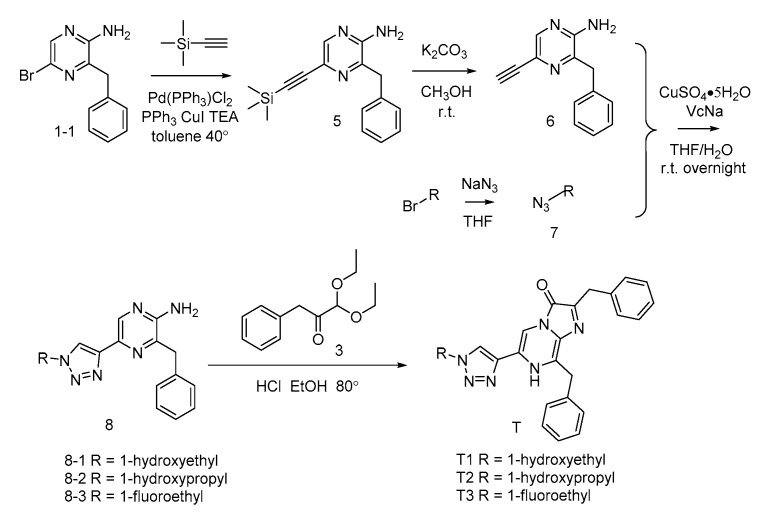
Synthesis of the T-series CTZ analogues.

For the T-series CTZ analogues (Figure 6) [15], the compound **1-1** underwent a Sonogashira coupling reaction with trimethylsilylacetylene and then became 2-amino-3-benzyl-5-ethynyl-pyrazine (**6**) by removing the trimethylsilyl group. Then, the key precursors **8** were synthesized using a click reaction of the compound **6** and azides.

The benzylketoacetal (**3**), which was required for the final condensation step, was synthesized by the Grignard reagent benzylmagnesium chloride and ethyl diethoxyacetate. Then, the coelenteramines **2**, **4**, and **8** were condensed with the compound **3** respectively, yielding the coelenterazine analogues of A-series, B-series and T-series, respectively.

Synthesis details are available in the Supplementary Materials.

### 3.3. Protein Preparation

For apo-obelin and apo-aequorin production, the *Escherichia coli* BL21-Gold (DE3) Codon Plus (RIPL) cells transformed with the corresponding plasmids [45,46] were cultivated with vigorous shaking at 37 °C in LB medium containing ampicillin (200 µg/mL). Protein expression was induced with 1 mM IPTG at OD_590_ 0.6–0.8 and the cultivation was continued for another 3 h. Recombinant apophotoproteins were extracted from *E. coli* inclusion bodies by 6 M urea as previously reported [47]. Apophotoproteins were purified on a DEAE-Sepharose Fast Flow column (GE Healthcare, Chicago, IL, USA), and then concentrated with the use of 10 kDa Ultra Centrifugal Filters (Merck KGaA, Darmstadt, Germany). The concentrations of apophotoproteins were determined using the corresponding molar extinction coefficients at 280 nm calculated with the ProtParam tool (http://us.expasy.org/tools/protparam-doc.html) that uses the method of Edelhoch [48].

The active photoproteins were produced by overnight incubation of apophotoprotein with a twofold molar excess of coelenterazine (or its analogue) in a buffer 5 mM EDTA, 10 mM DTT, 20 mM Tris-HCl pH 7.2 at 4 °C. Active photoprotein was separated from apophotoprotein, unbound coelenterazine, and DTT by ion-exchange chromatography on HiTrap Q HP column (GE Healthcare, Chicago, IL, USA). Active photoproteins and apophotoproteins were eluted as separate peaks with the linear salt gradient of 1 M NaCl in 5 mM EDTA, 20 mM Tris-HCl pH 7.2. Protein concentration was determined with the Dc Bio-Rad protein assay kit (Bio-Rad, Hercules, CA, USA).

### 3.4. Determination of the Specific Bioluminescence Activity

Specific bioluminescence activity of photoproteins activated with both native coelenterazine and its analogues was measured with a luminometer BLM-003 (Oberon-K, Krasnoyarsk, Russia) by rapid injection of 10 µL of photoprotein solution in 1 mM EDTA, 20 mM Tris-HCl pH 7.2 into a luminometer cell containing 490 µL of 2 mM CaCl_2_ in 50 mM Tris-HCl pH 8.8 at room temperature. In order to calculate specific bioluminescence activities, the bioluminescence signal was integrated over 10 s.

### 3.5. Spectral Measurements

Absorption spectra were obtained with an UV-2600 double-beam spectrophotometer (Shimadzu, Kyoto, Japan). Bioluminescence and fluorescence spectra were measured with a Cary Eclipse spectrofluorometer (Agilent, Santa Clara, CA, USA) in 1 mM EDTA, 20 mM Tris-HCl pH 7.0. The slit width was 5 nm. Bioluminescence was initiated by injecting CaCl_2_ solution into the same buffer. The concentration of free calcium was around 0.5 μM to provide a constant light level during the spectral scans. The fluorescence spectra of Ca^2+^-discharged photoproteins were recorded after the bioluminescence reaction ceased. Excitation wavelength was 350 nm. All bioluminescence and fluorescence spectra were corrected for the detector spectral sensitivity with an algorithm supplied with the instrument.

### 3.6. EDTA-Free Solutions of Photoproteins

EDTA was removed from the purified photoproteins by gel filtration on a 1.5 × 6.5 cm D-Salt Dextran Desalting column (Thermo Fisher Scientific, Waltham, MA, USA). The column was equilibrated and the protein was eluted with 150 mM KCl, 5 mM piperazine-1,4-bis(2-ethanesulfonic acid) (PIPES), pH 7.0 previously passed (twice) through freshly washed beds of Chelex 100 chelating resin (Merck KGaA, Darmstadt, Germany) to remove the trace amounts of Ca^2+^. The fractions containing photoproteins were identified by bioluminescence assay. To avoid possible contamination with EDTA, only the first few protein fractions to come off the column were used for rapid mixing measurements.

### 3.7. Stopped-Flow Measurements and Kinetic Analysis

Fast kinetics measurements were performed in EDTA-free solutions of the photoprotein. The light response kinetics after a sudden exposure to a saturating Ca^2+^ concentration was examined with an SX20 stopped-flow machine (cell volume 20 µL, dead time 1.1 ms) (Applied Photophysics, Leatherhead, UK). The temperature was controlled with a circulating water bath and was set at 20 °C in all experiments. The Ca^2+^ syringe contained 40 mM Ca^2+^, 30 mM KCl, 5 mM PIPES, pH 7.0. The 2 µM photoprotein was dissolved in a Ca^2+^-free solution of the same ionic strength: 150 mM KCl, 5 mM PIPES, pH 7.0. Both syringes were prewashed with the EDTA solution and then thoroughly washed with deionized water. The solutions were mixed in equal volumes. Thus, the final concentrations of Ca^2+^ and photoprotein in the reaction mixture were 20 mM and 1 µM, respectively.

Bioluminescence rise and decay rate constants were calculated by one- or two-exponential fitting using averaging of five independent shots. The rise rate constant was calculated by one-exponential fitting with Sigma Plot software in the range from zero time to the time the light signal reaches its maximum with the equation

(1) $$L=L_{0}+a(1-e^{-k_{rise}t})$$

The decay rate constants were calculated by two-exponential fitting. Contribution of the decay rate constants *k*_1_ and *k*_2_ was estimated as the relative amplitude calculated from the fitted amplitudes *a* and *b* with their sum normalized to 1 [49].

### 3.8. Calcium Concentration-Effect Curves

Measurements were performed in EDTA-free solutions of the photoproteins. For the 10^−6^ to 10^−2^ M [Ca^2+^] range, simple dilutions of CaCl_2_ were used and for Ca^2+^ concentrations below 10^−6^ M the Ca-EGTA buffers (total [EGTA] = 2 mM) were used. The Ca^2+^ buffers were prepared by the two stock-solution method described elsewhere [50]. Peak light intensity (L) was measured after the injection of 10 μL of photoprotein solution into 1 mL of the test solution. All measurements were carried out at 20 °C using a luminometer equipped with a temperature-stabilized cuvette block and neutral density filters with different transmission coefficients to fit light signals from low to saturated calcium concentrations ranging over 6–7 log units. Ca^2+^ concentration-effect curves were presented as L/L_int_ vs. [Ca^2+^], where L is the peak bioluminescence intensity for a given [Ca^2+^] value and L_int_ is the total bioluminescence recorded for a saturating Ca^2+^ dose for the same sample [39].

When measurements for Ca^2+^ concentration-effect curves were to be made in solutions containing magnesium, the photoprotein samples were pre-equilibrated for 1 h with 1 mM Mg^2+^. In this case, all other solutions (Ca–EGTA buffers, dilutions of CaCl_2_) also contained 1 mM Mg^2+^.

The Ca^2+^ concentration detection limit ([Ca^2+^]_limit_) was estimated as the Ca^2+^ concentration which will give a light signal five times the level of Ca^2+^-free luminescence and was calculated as the mean of the detection limits estimated from three Ca^2+^ concentration-effect curves for each photoprotein. The stated error is the standard deviation. The apparent dissociation constants for calcium (*K*_d_) were estimated from Ca^2+^ concentration-effect curves using a two-state model [13].

## 4. Conclusions

In this paper, we described the bioluminescent properties of semi-synthetic aequorins and obelins activated by novel coelenterazine analogues and the recently reported coelenterazine derivatives that were tested earlier with Renilla and Gaussia luciferases. Only a few of the novel semi-synthetic photoproteins retained moderate bioluminescence activities compared to the corresponding photoproteins with native CTZ.

All the T-series and most of the A-series analogues showed weak bioluminescence with aequorin or obelin, which indicated that the introduction of an alkynyl bond and triazole group at C-6 position interfere with the photoprotein bioluminescence reaction or promote undesirable conformational changes in protein structure. For the B-series, the introduction of electron-withdrawing groups such as nitrilephenyl or methoxyphenyl, or larger groups such as benzofuranyl, naphthyl or ethoxycarbonylphenyl, would also disturb photoprotein bioluminescence reaction. However, semi-synthetic photoproteins activated by the compound A6 or B14 (CTZ-*h*) had the best bioluminescent performance among all the studied variants. Only these two CTZ analogues were capable of inducing efficient bioluminescence both with aequorin and obelin. In addition, Aq_B15, Ol_B1, and Ol_B3 also displayed moderate bioluminescence compared to the photoproteins with native CTZ.

The results point to the importance of the OH-group at the C-6 phenyl ring of coelenterazine for bioluminescence emission since it can promote the interaction with the surrounding amino acid residues and formation of hydrogen bond with tyrosine residue of aequorin. Moreover, the location and the number of OH-groups at C-6 phenyl ring of the CTZ imidazopyrazinone core seems to be of the extreme significance as both semi-synthetic aequorin and obelin activated by the coelenterazine analogue with three OH-groups at the C-6 phenyl ring of coelenterazine display poor bioluminescence activities [24]. At the same time, the CTZ analogues with 4-fluorophenyl or 4-hydroxymethylphenyl at C-6 position (compounds B1 and B3, respectively) could still interact with the adjacent Phe of obelin. Thus, the substituent at position 6 of the CTZ imidazopyrazinone core was shown to play an important role in the interactions with certain amino acid residues of bioluminescent proteins and consequently to be crucial for the photoprotein bioluminescence, while the presence of a triple bond or the absence of the hydroxy group in 2-benzyl ring of CTZ apparently was not essential for the bioluminescence reaction of photoproteins.

Although some of the semi-synthetic obelins with weak bioluminescence had shifted spectra, semi-synthetic photoproteins with high bioluminescence activities did not show any promising spectral shifts. Meanwhile, the semi-synthetic photoproteins activated by the above-mentioned A6, B1, and B14 demonstrated higher Ca^2+^ sensitivity compared to that of the corresponding photoproteins activated by native CTZ, which points to their potential as Ca^2+^ sensors. Aq_A6 displayed faster kinetics compared to Aq_WT, which can be useful for certain applications.

On the whole, the A6 coelenterazine derivative has displayed the best performance at being applied as a substrate of photoprotein bioluminescence with both aequorin and obelin. Aq_A6 and Ol_A6 preserved sufficient bioluminescence activities (79% and 70%, respectively) as compared to those of the corresponding photoproteins with native CTZ. At the same time, spectral properties as well as calcium sensitivity remained close to those of Aq_WT and Ol_WT. Thus, the A6 compound can serve as a basis for synthesizing new coelenterazine analogues suitable for the activation of photoproteins.

Previous studies on A-, B-, and T-series of coelenterazine analogues showed that RLuc with B2 and B9 compounds exhibited 90% and 25% of the bioluminescence activity with native CTZ, respectively [15,16]. However, GLuc displayed poor bioluminescence signals with most of the studied coelenterazine analogues including B2, which demonstrated 5% of the GLuc bioluminescence activity with native CTZ [15,16]. Both aequorin and obelin also revealed low bioluminescence activity with these coelenterazine analogues as compared to the Aq_WT and Ol_WT.

It is important to note that the amino acid environment of the active sites of Ca^2+^-regulated photoproteins, being quite different from that of Renilla and Gaussia luciferases, may account for low bioluminescence activity of the semi-synthetic photoproteins tested. Moreover, semi-synthetic obelins and aequorins displayed different properties on being activated by the same coelenterazine analogue despite the fact that the substrate-binding cavity of these photoproteins is formed by the strictly conserved amino acid residues. For example, 4-fluorophenyl and 4-hydroxymethylphenyl substituents at the 6-position of imidazopyrazinone core were not an obstacle for coelenterazine in forming an active photoprotein complex with obelin, unlike what happened with aequorin. This strictly means that the amino acid environment of the coelenterazine molecule bound within the active site of a bioluminescent protein could be a determining factor in the bioluminescence efficiency of certain coelenterazine analogues. The experiments on F88Y obelin and Y82F aequorin mutants activated by B1 and B15 CTZ analogues confirmed that the hydrogen-bond network and other possible interactions of the residues surrounding the substituent at position 6 of the CTZ imidazopyrazinone core influence both light emission color and efficiency of bioluminescence reaction.

Overall, by summarizing the effects of substrate modifications on the properties of Ca^2+^-regulated photoproteins the present study provides helpful information for further research and development of the Ca^2+^-regulated semi-synthetic photoproteins.

## Figures and Tables

**Table 1:** Bioluminescent properties of semi-synthetic aequorins and obelins.

CTZ	Aequorin			Obelin		
	Activity %	Bioluminescence λ_max_ (nm)	Fluorescence λ_max_ (nm)	Activity %	Bioluminescence λ_max_ (nm)	Fluorescence λ_max_ (nm)
native	100.00	473	470	100.00	390/**482**	505
A1	0.40	468	407	8.2	390/**475**	410
A2	0.20	446	441	0.02	434	**410**/510
A3	0.04	458	407	0.01	432	**407**/500
A4	0.03	466	407	0.01	444	**404**/500
A5	0.03	466	407	0.01	444	**404**/500
A6	79.0	468	476	70.0	390/**478**	512
B1	0.20	470	422	48.0	390/**476**	420/**512**
B2	0.50	452	488	5.3	432	437
B3	0.84	468	422	22.7	390/**476**	420/**512**
B4	0.25	478	416	6.6	390/**477**	**410**/508
B6	0.16	475	420	0.2	390/**475**	416
B7	0.02	ND	**422**/505	0.25	390/**477**	**420**/505
B8	0.02	ND	430	0.06	ND	423
B9	0.06	ND	420	0.10	390/**477**	428
B10	0.06	ND	430	0.06	ND	428
B11	0.07	473	**430**/510	0.05	**390**/482	**411**/518
B13	0.01	ND	414	0.04	ND	**409**/510
B14	50.0	470	400/**464**	76.1	482	410/**501**
B15	30.0	470	400/**480**	2.5	390/**478**	410/**500**
B16	0.70	473	424/**484**	0.09	474	440–490
T1	0.08	473	416	0.08	390/**480**	416
T2	0.09	470	413	0.02	ND	413
T3	0.02	ND	416	0.01	ND	416

ND—not detected; for bimodal spectra λ_max_ is shown in bold. Bioluminescence activity was measured as the total light emitted until the reaction ceased and calculated using averaging of three independent measurements. Fluorescence spectra were taken from the Ca^2+^-discharged photoproteins, which are the photoproteins after bioluminescence reaction. Excitation wavelength was 350 nm. Spectral maxima were calculated using averaging of 10 independent spectra in the case of bioluminescence and 5 independent spectra in the case of fluorescence.

**Table 2:** Bioluminescence activities of F88Y obelin and Y82F aequorin mutants with certain coelenterazine analogues.

Protein	Bioluminescence Activity, %		
	Native CTZ	B1	B15
Obelin WT	100	48.0	2.5
Obelin F88Y	100	16.0	19.0
Aequorin WT	100	0.2	30.0
Aequorin Y82F	100	45.0	1.2

**Table 3:** Absorbance spectra maxima of coelenterazine analogues in ethanol, and active and Ca^2+^-discharged semi-synthetic aequorin and obelin.

CTZ	Absorbance (λ_max_, nm)				
	In Ethanol	Aequorin		Obelin	
		Active	Ca^2+^-Discharged	Active	Ca^2+^-Discharged
**native**	350/**434**	460	335	460	335
A1	**344**/405	344	344	460	335
A2	**386**/434	344	344	ND	ND
A3	**393**/434	344	344	ND	ND
A4	**390**/434	344	344	344	344
A5	**386**/434	350	350	350	350
A6	**386**/434	460	335	460	344
B1	358/**434**	**338**/405	**338**/405	**344**/460	344
B2	434	**350**/400	340/430	**350**/430	**350**/430
B3	358/**434**	**350**/410	**350**/410	**340**/420	**345**/420
B4	**334**/394	336	336	**336**/445	**336**/445
B6	**330**/434	322	322	**360**/445	**360**/445
B7	**344**/422	**314**/400	425	346	346
B8	395	**350**/400	**350**/400	350	350
B9	**350**/418	**350**/400	**350**/400	**360**/425	**360**/425
B10	354/**437**	**352**/400	**352**/400	360	360
B11	**354**/426	**354**/426	**354**/426	**335**/365	433
B13	354/**430**	330	330	334	**334**/427
B14	344/**434**	**344**/434	344	**344**/434	344
B15	**380**/450	400	**320**/417	377	**320**/387
B16	390	370	370	380	380
T1	**349**/428	**334**/400	**334**/400	**334**/400	**334**/400
T2	**344**/426	**334**/400	**334**/400	**334**/400	**334**/400
T3	349/**430**	**334**/400	**334**/400	**334**/400	**334**/400

Active photoprotein—photoprotein before bioluminescence reaction; Ca^2+^-discharged photoprotein—photoprotein after bioluminescence reaction. ND—not detected; for bimodal spectra λ_max_ is shown in bold.

**Table 4:** Calcium sensitivity parameters of semi-synthetic and wild type photoproteins.

Photoprotein	[Ca^2+^]_limit_, nM		*K*_d_, nM	
	Without Mg^2+^	With 1 mM Mg^2+^	Without Mg^2+^	With 1 mM Mg^2+^
Ol_WT	52 ± 4	57 ± 5	87 ± 11	112 ± 9
Ol_A6	40 ± 5	48 ± 4	71 ± 8	100 ± 8
Ol_B1	43 ± 5	63 ± 6	74 ± 9	105 ± 10
Ol_B14	21 ± 2	23 ± 2	33 ± 2	45 ± 5
Aq_WT	19 ± 2	53 ± 5	41 ± 5	100 ± 9
Aq_A6	27 ± 4	46 ± 5	38 ± 3	83 ± 6
Aq_B14	5.6 ± 1	12 ± 1	6.3 ± 3	33 ± 4

**Table 5:** Rapid-mixing kinetics of semi-synthetic photoproteins.

Photoprotein	*k*_rise_, s^−1^	*k*_decay_	
		*k*_1_, s^−1^	*k*_2_, s^−1^
Ol_WT	510.0 ± 5.0	40.0 ± 1.75 (0.66)	4.8 ± 0.01 (0.34)
Ol_A6	599.3 ± 7.7	38.1 ± 0.14 (0.62)	5.9 ± 0.01 (0.38)
Ol_B1	677.1 ± 14.2	39.2 ± 0.29 (0.44)	5.0 ± 0.01 (0.56)
Ol_B14	577.6 ± 30.5	38.5 ± 0.14 (0.60)	5.5 ± 0.01 (0.40)
Aq_WT	123.0 ± 1.0	0.81 ± 0.01	-
Aq_A6	302.9 ± 3.4	8.11 ± 0.03 (0.37)	0.86 ± 0.01 (0.63)
Aq_B14	160.3 ± 1.7	1.17 ± 0.01	-

Contribution of decay rate constants *k*_1_ and *k*_2_ was estimated as the relative amplitude calculated from the fitted amplitudes *a* and *b* with their sum normalized to 1 (in parentheses).

## Supplementary Materials

Supplementary Materials can be found at https://www.mdpi.com/1422-0067/21/15/5446/s1.

## Abbreviations

RLuc	luciferase from *Renilla reniformis*
GLuc	luciferase from *Gaussia princeps*
CTZ	coelenterazine
Ol	obelin from *Obelia longissima*
Aq	aequorin from *Aequorea victoria*
Ol_WT	obelin activated by native coelenterazine
Ol_A1	obelin activated by A1 coelenterazine analogue
Ol_A2-A5	obelins activated by A2, A3, A4, A5 analogues
Ol_A6	obelin activated by A6 coelenterazine analogue
Ol_B1	obelin activated by B1 coelenterazine analogue
Ol_B2	obelin activated by B2 coelenterazine analogue
Ol_B3	obelin activated by B3 coelenterazine analogue
Ol_B4	obelin activated by B4 coelenterazine analogue
Ol_B11	obelin activated by B11 coelenterazine analogue
Ol_B14	obelin activated by B14 coelenterazine analogue
Aq_WT	aequorin activated by native coelenterazine
Aq_A1	aequorin activated by A1 coelenterazine analogue
Aq_A6	aequorin activated by A6 coelenterazine analogue
Aq_B1	aequorin activated by B1 coelenterazine analogue
Aq_B2	aequorin activated by B2 coelenterazine analogue
Aq_B14	aequorin activated by B14 coelenterazine analogue
Aq_B15	aequorin activated by B15 coelenterazine analogue
EDTA	ethylenediaminetetraacetic acid
EGTA	ethyleneglycol-bis(β-aminoethyl ether)tetraacetic acid
IPTG	isopropyl β-d-1-thiogalactopyranoside
DEAE	diethylaminoethyl (cellulose)
DTT	dithiothreitol
PIPES	piperazine-1,4-bis(2-ethanesulfonic acid)
