# Supplementary material for: Bioluminescent Properties of Semi-Synthetic Obelin and Aequorin Activated by Coelenterazine Analogues with Modifications of C-2, C-6, and C-8 Substituents
Source: Int J Mol Sci. 2020 Jul 30;21(15):5446. doi: 10.3390/ijms21155446 (PMC7432523; doi:10.3390/ijms21155446)
Supplement: Supplementary file 1 [file ijms-21-05446-s001.pdf]

Supplementary

Bioluminescent properties of semi-synthetic obelin and aequorin activated by coelenterazine analogues with modifications of C-2, C-6, and C-8 substituents

Organic synthesis of A-series 2

Organic synthesis of B-series 5

Organic synthesis of T-series 10

Bioluminescence spectra 103

Fluorescence spectra 103

Absorption spectra 104

Organic synthesis of A-series

The preparation of 3-benzyl-5-bromo-2-amino-pyrazine (**1-1**):

Zn dust (235 mg, 3.6 mmol) and I_2 (12 mg) were suspended in fresh anhydrous THF under argon atmosphere and the mixture was stirred at room temperature until the brown color of I_2 disappeared. Then the anhydrous benzyl bromide was added by using a syringe and the reaction mixture was refluxed at 80°C for 3 h. After insertion of Zn, the reaction mixture was cooled to room temperature. Then the suspension of 2-amino-3, 5-dibromopyrazine (506 mg, 2 mmol) and $PdCl_2(PPh_3)_2$ (70 mg, 0.1 mmol) in 7 mL of DMF was added. The reaction mixture was continuously stirred overnight. Then the mixture was filtered by celatom and the filtrate was collected and evaporated under vacuum. The collection was dissolved in and extracted by ethyl acetate and washed with saturated sodium chloride solution. After dried over anhydrous sodium sulfate and concentrated under reduced pressure, the crude product was further purified by chromatography on silica gel (PE/EtOAC 20:3) to give viscous yellow solid (313 mg). Yield: 59%. ^{1}HNMR (400 MHz, DMSO-d6): δ 7.65 (s, 1 H), 7.30~7.25 (m, 5 H), 6.56 (s, 1 H), 3.98(s, 2H). ESI-MS: m/z [M+H] + calcd for 264.01, 266. 01, found 264.2, 266.3.

The preparation of 2-amino-3-benzyl-5-phenylethynyl -pyrazine (**2-1**):

The suspension of $PdCl_2(PPh_3)_2$ (27 mg, 0.038 mmol), CuI (29 mg, 0.151 mmol) and PPh_3 (20 mg, 0.076 mmol) in 2 mL toluene was added to the solution of 3-benzyl-5-bromo-2-amino-pyrazine (200 mg, 0.76 mmol) in 3 mL toluene under argon atmosphere. Then phenylacetylene (232 mg, 2.27 mmol) and triethylamine (0.7 mL) were sequentially added. The mixture was stirred at 40°C for 6 h. Then the mixture was filtered and the filtrate was collected and evaporated under vacuum. The collection was extracted by ethyl acetate and washed with saturated sodium chloride solution. After dried over anhydrous sodium sulfate and concentrated under reduced pressure, the crude product was further purified by chromatography on silica gel (PE/EtOAC 10:1) to give yellow solid (165 mg). Yield: 77%. ^{1}HNMR (400 MHz, DMSO-d6): δ8.09(s, 1H), 7.43~7.41(m, 6H), 7.29~7.28(m, 4H), 6.81(s, 2H), 4.04(s, 2H). ESI-MS: m/z $[M+H]^+$ calcd for 286.13, found 286.4.

2-2, **2-3**, **2-4**, **2-5** and **2-6** were prepared with similar method.

2-Amino-3-benzyl-5-(4-fluorophenylethynyl)-pyrazine (**2-2**): yellow solid; yield 50%; ^{1}HNMR (400 MHz, DMSO-d6): δ8.09(s, 1H), 7.61~7.58(m, 2H), 7.48~7.43(m, 2H), 7.30~7.21(m, 5H). 6.82(s, 2H), 4.03(s, 2H). ESI-MS: m/z $[M+H]^+$ calcd for 304.13, found 304.1.

2-Amino-3-benzyl-5-(3-fluorophenylethynyl)-pyrazine (**2-3**): yellow solid; yield 45%; ^{1}HNMR (400 MHz, DMSO-d6): δ8.11(s, 1H), 7.49~7.38(m, 3H), 7.32~7.19(m, 6H), 6.86(s, 2H), 4.04(s, 2H). ESI-MS: m/z $[M+H]^+$ calcd for 304.13, found 304.4.

2-Amino-3-benzyl-5-(2-fluorophenylethynyl)-pyrazine (**2-4**): yellow solid; yield 44%; ^{1}HNMR (400 MHz, DMSO-d6): δ8.11(s, 1H), 7.64~7.60(td, J=7.53Hz, 1.6Hz, 1H), 7.48~7.46(m, 1H), 7.36~7.20(m, 7H), 6.88(s, 2H), 4.04(s, 2H). $[M+H]^+$ calcd for 304.13, found 304.4.

2-Amino-3-benzyl-5-(3-methylphenylethynyl)-pyrazine (**2-5**): yellow solid; yield 62%; ^{1}HNMR (400 MHz, DMSO-d6): δ8.07(s, 1H), 7.36~7.19(m, 9H), 6.77(s, 2H), 4.03(s, 2H), 2.31(s, 3H). ESI-MS: m/z $[M+h]^+$ calcd for 300.15, found 300.5.

2-Amino-3-benzyl-5-(3-hydroxyphenylethynyl)-pyrazine (**2-6**): yellow solid; yield 62%; ^{1}HNMR (400 MHz, DMSO-d6): δ9.69(s, 1H), 8.07(s, 1H), 7.29~7.26(m, 3H), 7.22~7.18(t, J=8Hz, 2H), 6.95(d, J=8Hz, 1H), 6.87(s, 1H), 6.82~6.79(m, 1H), 6.77(s, 2H), 4.03(s, 2H). ESI-MS: m/z $[M+H]^+$ calcd for 302.13, found 302.5.

The preparation of 3-phenyl-1,1-diethoxyacetone (**3**):

Ethyl diethoxyacetate (500 mg, 2.84 mmol) was dissolved in fresh anhydrous THF and the solution was cooled to -78°C under argon atmosphere. Then benzylmagnesium chloride (642 mg, 4.26 mmol) solution was added via syringe over 15 min and the reaction was allowed to stir for 3 h. The reaction was quenched by addition of ammonium chloride aqueous solution and then allowed to warm to room temperature. The reagent was evaporated under vacuum and redissolved in ethyl acetate. After extraction by ethyl acetate, the organic layer was washed with saturated sodium chloride aqueous solution. The mixture was concentrated under reduced pressure and then was subjected to chromatography on silica gel (PE/EtOAC 20:1) to give colorless oil (380 mg). Yield: 60%. ^{1}HNMR (400 MHz, DMSO-d6): δ7.31~7.16 (m, 6H), 4.80(s, 1H) 3.87 (s, 2 H), 3.66~3.53 (m, 4H). ESI-MS: m/z $[M+NH_4]^+$ calcd for 240.16, found 240.5.

The preparation of 2-benzyl-8-benzyl-6-(phenylethynyl)imidazo[1,2-a]pyrazin-3(7*H*)-one (**A1**):

The mixture of 2-amino-3-benzyl-5-phenylethynyl-pyrazine (4-1, 80 mg, 0.28 mmol) and 3-phenyl - 1,1-diethoxyacetone (3-1, 124 mg, 0.56 mmol) was dissolved in ethanol (3 mL) under argon atmosphere and allowed to stir at room temperature for 10 min. The con.HCl (0.2 mL) in ethanol (1 mL) was then added to the mixture via syringe over 10 min. The reaction was heated to refluxed at 80°C for 8 h and then allowed to cool to room temperature. The crude was concentrated under vacuum and further purified by chromatography on silica gel (CH_2Cl_2/MeOH 50:1) to give light brown solid (30 mg). Yield: 26%. Analytical RP HPLC (Phenomenex, C8, 250 x 4.6 mm column): 60% acetonitrile with 0.1% trifluoroacetic acid, 1.0 mL/min at 380 nm, Rt: 7.448 min, 97%. ^{1}HNMR (400 MHz, CD_3OD): δ8.47(s, 1H), 7.58~7.24(m, 15H), 4.59(s, 2H), 4.34(s, 2H). ^{13}CNMR (100 MHz, CD_3OD): δ147.24, 137.12, 134.94, 131.58, 129.75, 128.94, 128.60, 128.45, 128.42, 127.28, 126.60, 120.68, 117.41, 94.22, 80.17, 35.89, 31.07. ESI-HRMS: m/z $[M+H]^+$ calcd for 416.1763, found 416.1757.

A2, **A3**, **A4**, **A5** and **A6** were prepared with similar method.

2-Benzyl-8-benzyl-6-(4-fluorophenylethynyl)imidazo[1,2-a]pyrazin-3(7*H*)-one (**A2**): yellow solid; yield 35%. Analytical RP HPLC (Phenomenex, C8, 250 x 4.6 mm column): 60% acetonitrile with 0.1% trifluoroacetic acid, 1.0 mL/min at 380 nm, Rt: 7.842 min, 96%. ^{1}HNMR (400 MHz, CD_3OD): δ8.07(s, 1H), 7.64~7.60(m, 2H), 7.38~7.36(m, 2H), 7.29~7.21(m, 7H), 7.19~7.15(m, 3H), 4.42(s, 2H), 4.22(s, 2H). ^{13}CNMR (100 MHz, CD_3OD): δ164.74, 162.25, 147.35, 140.52, 137.03, 134.98, 134.07, 134.03, 133.98, 128.85, 128.38, 128.34, 127.23, 127.10, 126.57, 117.39, 115.84, 115.61, 92.98, 80.05, 35.85, 30.94. ESI-HRMS: m/z $[M+H]^+$ calcd for 434.1669, found

434.1663.

2-Benzyl-8-benzyl-6-(3-fluorophenylethynyl)imidazo[1,2-a]pyrazin-3(7*H*)-one (**A3**): yellow solid; yield 30%. Analytical RP HPLC (Phenomenex, C8, 250 x 4.6 mm column): 60% acetonitrile with 0.1% trifluoroacetic acid, 1.0 mL/min at 370 nm, Rt: 6.173 min, 97%. ^{1}HNMR (400 MHz, CD_3OD): δ8.58(s, 1H), 7.46~7.20(m, 14H), 4.59(s, 2H), 4.34(s, 2H). ^{13}CNMR (100 MHz, CD_3OD): δ163.64, 161.19, 147.39, 137.00, 134.98, 130.58, 130.49, 128.56, 128.40, 128.34, 127.75, 127.22, 126.60, 118.19, 117.95, 117.72, 117.04, 116.82, 92.29, 81.25, 35.98, 30.83. ESI-HRMS: m/z $[M+H]^+$ calcd for 434.1669, found 434.1664.

2-Benzyl-8-benzyl-6-(2-fluorophenylethynyl)imidazo[1,2-a]pyrazin-3(7*H*)-one (**A4**): yellow solid; yield 14%. Analytical RP HPLC (Phenomenex, C8, 250 x 4.6 mm column): 65% acetonitrile with 0.1% trifluoroacetic acid, 1.0 mL/min at 380 nm, Rt: 5.788 min, 97%. ^{1}HNMR (400 MHz, CD_3OD): δ8.57(s, 1H), 7.69~7.65(td, J=7.36Hz, 1.6Hz, 1H), 7.52~7.43(m, 3H), 7.34~7.22(m, 10H), 4.59(s, 2H), 4.34(s, 2H). ^{13}CNMR (100 MHz, CD_3OD): δ164.06, 161.55, 147.41, 140.59, 136.99, 134.98, 133.59, 128.80, 128.40, 128.31, 127.21, 126.60, 124.42, 124.39, 117.71, 115.55, 15.34, 109.38, 109.22, 87.15, 85.15, 35.96, 30.82. ESI-HRMS: m/z $[M+H]^+$ calcd for 434.1669, found 434.1662.

2-Benzyl-8-benzyl-6-(3-methylphenylethynyl)imidazo[1,2-a]pyrazin-3(7*H*)-one (**A5**): yellow solid; yield 40%. Analytical RP HPLC (Phenomenex, C8, 250 x 4.6 mm column): 65% acetonitrile with 0.1% trifluoroacetic acid, 1.0 mL/min at 380 nm, Rt: 6.910 min, 95%. ^{1}HNMR (400 MHz, CD_3OD): δ7.70(s, 1H), 7.40~7.18(m, 14H), 4.34(s, 2H), 4.18(s, 2H), 2.36(s, 3H). ^{13}CNMR (100 MHz, CD_3OD): δ147.24, 140.63, 138.54, 138.47, 137.10, 134.99, 134.95, 134.02, 131.94, 130.95, 130.58, 128.86, 128.79, 128.72, 128.56, 128.37, 128.22, 128.12, 127.24, 126.56, 120.95, 120.57, 117.28, 94.50, 79.72, 35.73, 31.06, 20.1. ESI-HRMS: m/z $[M+H]^+$ calcd for 430.1919, found 430.1914.

2-Benzyl-8-benzyl-6-(3-hydroxyphenylethynyl)imidazo[1,2-a]pyrazin-3(7*H*)-one (**A6**): yellow solid; yield 15%. Analytical RP HPLC (Phenomenex, C8, 250 x 4.6 mm column): 60% acetonitrile with 0.1% trifluoroacetic acid, 1.0 mL/min at 370 nm, Rt: 4.946 min, 99%. ^{1}HNMR (400 MHz, CD_3OD): δ7.69(s, 1H), 7.41~7.18(m, 14H), 4.33(s, 2H), 4.18(s, 2H). ^{13}CNMR (100 MHz, CD_3OD): δ157.37, 147.15, 137.09, 134.96, 129.59, 128.80, 128.57, 128.36, 127.25, 126.56, 122.87, 121.52, 117.94, 117.28, 94.44, 79.38, 35.66, 31.11. ESI-HRMS: m/z $[M+H]^+$ calcd for 432.1712, found 432.1701.

Organic synthesis of B-series

The preparation of 2-amino-3-(5-methylfuryl-2)-5-bromo-pyrazine (**1-2**):

2-amino-3, 5-dibromopyrazine (500 mg, 1.98 mmol) was added to a suspension of Pd(dppb)Cl_2 (68 mg, 0.11 mmol) and $(C_6H_5CN)_2PdCl_2$ (43 mg, 0.11 mmol) in toluene (6 mL) and stirred at room temperature under argon atmosphere. 4,4,5,5-tetramethyl-2-(5-methylfuran-2-yl)-1,3,2-dioxaborolane (534 mg, 2.57 mmol) in toluene (4 mL) and then potassium carbonate aqueous solution (2 M, 0.6 mL) was sequentially added to this mixture with stirring. The mixture was heated to reflux at 109°C for 8 h and then allowed to cool to room temperature. The mixture was evaporated under vacuum and redissolved in ethyl acetate. Then it was extracted by ethyl acetate and washed with

saturated sodium chloride aqueous solution. After being dried over anhydrous sodium sulfate and concentrated under reduced pressure, the crude product was further purified by chromatography on silica gel (PE/EtOAC10:1) to give light yellow solid (300 mg). Yield: 60%. ^{1}HNMR (400 MHz, DMSO-d_6): δ 8.02 (s, 1H), 7.03 (d, J = 4Hz, 1H), 6.69 (s, 2H), 6.33 (dd, J = 4, 0.8Hz, 1H), 2.41 (s, 3H). ^{13}CNMR (100 MHz, DMSO-d_6): δ 154.2, 150.3, 148.6, 142.0, 129.9, 124.0, 113.1, 109.1, 14.0. ESI-MS: m/z $[M+H]^+$ calcd for 254.0, 256.0, found 254.1, 256.1.

The preparation of 2-amino-3-benzyl-5-(4-fluorophenyl)-pyrazine (**4-1**):

3-benzyl-5-bromo-2-amino-pyrazine (**1-1**, 500 mg, 1.89 mmol) was added to a suspension of Pd(dppb)Cl_2 (68 mg, 0.11 mmol) and $(C_6H_5CN)_2PdCl_2$(43 mg, 0.11 mmol) in toluene (6 mL) and stirred at room temperature under argon atmosphere. 4-fluorobenzeneboronic acid (397 mg, 2.84 mmol) in toluene (4 mL) and then potassium carbonate aqueous solution (2 M, 0.6 mL) was sequentially added to this mixture with stirring. The mixture was heated to reflux at 109°C for 8 h and then allowed to cool to room temperature. The mixture was evaporated under vacuum and redissolved in ethyl acetate. Then it was extracted by ethyl acetate and washed with saturated sodium chloride aqueous solution. After being dried over anhydrous sodium sulfate and concentrated under reduced pressure, the crude product was further purified by chromatography on silica gel (PE/EtOAC 20:3) to give white solid (395 mg). Yield: 75%. ^{1}HNMR (400 MHz, DMSO-d6): δ 8.42 (s, 1 H), 7.97~7.93 (m, 2 H), 7.3~7.18(m, 7H) 6.48 (s, 2 H), 4.09 (s, 2H). ^{13}CNMR (100 MHz, DMSO): δ163.5, 161.1, 152.9, 140.9, 138.5, 138.3, 134.1, 129.4, 128.7, 127.2, 127.1, 126.7, 116.1, 115.8, 39.0. ESI-HRMS: m/z $[M+H]^+$ calcd for 280.1250, found 280.1253.

4-2, **4-3**, **4-4**, **4-6**, **4-7**, **4-8**, **4-9**, **4-10**, **4-11, 4-13** and **4-14** were prepared with similar method.

2-Amino-3-benzyl-5-(4-amino-3-fluorophenyl)-pyrazine (**4-2**): yellow solid; yield 60%; ^{1}HNMR (400 MHz, DMSO-d6): δ8.31(s, 1H), 7.54~7.46(m, 2H), 7.34~7.26 (m, 4H), 7.21~7.17(m, 1H), 6.80~6.75(t, J=10Hz, 2H), 6.21(s, 2H), 5.26 (s, 2 H), 4.04 (s, 2H). ^{13}CNMR (100 MHz, DMSO): δ152.4, 140.1, 138.7, 136.5, 136.3, 136.2, 129.4, 128.7, 126.6, 121.4, 116.7, 116.6, 111.8, 111.6, 39.1. ESI-HRMS: m/z $[M+H]^+$ calcd for 295.1359, found 295.1340.

2-Amino-3-benzyl-5-(4-hydroxymethylphenyl)-pyrazine (**4-3**): yellow solid; yield 64%; ^{1}HNMR (400 MHz, DMSO-d6): δ8.41(s, 1H), 7.87(d, J=8Hz, 2H), 7.35 (d, J=8Hz, 4H), 7.28(t, J=8Hz, 2H), 7.21~7.17(t, J=8Hz, 1H), 6.37(s, 2H), 5.19 (t, J=6Hz, 1H), 4.51(d, J=8Hz, 2H), 4.08 (s, 2H). ^{13}CNMR (100 MHz, DMSO): δ153.1, 142.2, 140.4, 139.3, 138.6, 137.3, 136.1, 129.4, 128.7, 127.2, 126.6, 125.0, 63.1, 39.1. ESI-HRMS: m/z $[M+H]^+$ calcd for 292.1450, found 292.1454.

2-Amino-3-benzyl-5-(4-nitrilephenyl)-pyrazine (**4-4**): yellow solid; yield 54%; ^{1}HNMR (400 MHz, CD_3OD): δ8.45(s, 1H), 8.11(d, J=8Hz, 2H), 7.76 (d, J=8Hz, 2H), 7.32~7.23(m, 5H), 4.16 (s, 2H). ^{13}CNMR (100 MHz, DMSO): δ152.7, 142.7, 141.6, 138.0, 136.6, 133.2, 129.5, 128.8, 126.8, 125.6, 119.5, 110.1, 38.9. ESI-HRMS: m/z $[M+H]^+$ calcd for 287.1297, found 287.1299.

2-Amino-3-benzyl-5-(furyl-3)-pyrazine (**4-6**): yellow solid; yield 76%; ^{1}HNMR (400 MHz, DMSO-d6): δ8.19(s, 1H), 8.07(s, 1H), 7.70(s, 1H), 7.32~7.19(m, 5H), 6.91(s, 1H), 6.27(s, 1H), 4.04 (s, 2H). ^{13}CNMR (100 MHz,

DMSO): δ152.7, 144.5, 140.8, 139.5, 138.5, 136.9, 134.6, 129.3, 128.7, 126.6, 125.0, 108.6, 38.9. ESI-HRMS: m/z $[M+H]^+$ calcd for 252.1137, found 252.1141.

2-Amino-3-benzyl-5-(3-methoxycarbonylphenyl)-pyrazine (**4-7**): yellow solid; yield 74%; ^{1}HNMR (400 MHz, DMSO-d6): δ8.50(d, J=4Hz, 2H), 8.19(d, J=8Hz, 1H), 7.88(d, J=8Hz, 1H), 7.56(t, J=8Hz, 1H), 7.31(m, 4H), 6.55(s, 2H), 4.11(s, 2H), 3.88(s, 3H). ^{13}CNMR (100 MHz, DMSO): δ166.7, 153.6, 140.7, 138.4, 138.2, 137.9, 137.8, 130.6, 129.6, 129.4, 128.7, 128.3, 126.7, 125.7, 52.7, 39.0. ESI-MS: m/z $[M+H]^+$ calcd for 320.1399, found 320.1408.

2-Amino-3-benzyl-5-(benzofuranyl-2)-pyrazine (**4-8**): yellow solid; yield 37%; ^{1}HNMR (400 MHz, DMSO-d6): δ8.41(s, 1H), 7.65~7.59(m, 2H), 7.36~7.18(m, 7H), 6.72(s, 2H), 4.11 (s, 2H). ^{13}CNMR (100 MHz, DMSO): δ154.9, 154.6, 153.8, 141.5, 138.2, 137.5, 132.1, 129.3, 129.2, 128.8, 126.8, 124.7, 123.7, 121.4, 111.5, 101.5, 39.0. ESI-HRMS: m/z $[M+H]^+$ calcd for 302.1293, found 302.1293.

2-Amino-3-benzyl-5-(furyl-2)-pyrazine (**4-9**): yellow solid; yield 13%; ^{1}HNMR (400 MHz, DMSO-d6): δ8.21(s, 1H), 7.69(s, 1H), 7.32~7.18(m, 5H), 6.74(d, J=3.2Hz, 1H), 6.56(d, J=1.2Hz, 1H), 6.45(s, 2H), 4.06 (s, 2H). ^{13}CNMR (100 MHz, DMSO): δ153.0, 152.6, 142.9, 140.9, 138.3, 136.0, 133.2, 129.2, 128.7, 126.7, 112.3, 105.7, 38.9. ESI-HRMS: m/z $[M+H]^+$ calcd for 252.1137, found 252.1138.

2-Amino-3-benzyl-5-(4-methoxyphenyl)-pyrazine (**4-10**): yellow solid; yield 36%; ^{1}HNMR (400 MHz, $CDCl_3$): δ8.34(s, 1H), 7.90~7.87(d, *J*=12Hz, 2H), 7.34~7.24 (m, 5H), 7.01~6.98(d, *J*=12Hz, 2H), 4.35(s, 2H) 4.18 (s, 2 H), 3.86 (s, 3H). ^{13}CNMR (100 MHz, DMSO): δ159.4, 152.7, 136.6, 129.4, 128.7, 126.6, 126.5, 114.5, 55.6, 39.1. ESI-HRMS: m/z $[M+H]^+$ calcd for 292.1450, found 292.1452.

2-Amino-3-benzyl-5-naphthyl-pyrazine (**4-11**): light brown solid; yield 17%; ^{1}HNMR (400 MHz, DMSO-d6): δ8.60(s, 1H), 8.45(s, 1H), 8.12(d, J=8Hz, 1H), 7.95(d, J=8Hz, 2H), 7.90(d, J=8Hz, 1H), 7.54~7.48(m, 2H), 7.38(d, J=4Hz, 2H), 7.31(t, J=8Hz, 2H), 7.21(t, J=8Hz, 1H), 6.55(s, 2H), 4.15 (s, 2H). ^{13}CNMR (100 MHz, DMSO): δ153.3, 140.7, 139.0, 138.6, 137.8, 135.1, 133.7, 132.8, 129.4, 128.7, 128.6, 128.6, 128.0, 126.8, 126.7, 126.3, 123.8, 123.4, 39.1. ESI-HRMS: m/z $[M+H]^+$ calcd for 312.1501, found 312.1497.

2-Amino-3-benzyl-5-phenyl-pyrazine (**4-13**): light brown solid; yield 70%; ^{1}HNMR (400 MHz, $CDCl_3$): δ8.40 (s, 1H), 7.95(d, J=8Hz, 2H), 7.46(t, J=8Hz, 2H), 7.38~7.26(m, 6H), 4.41(s, 2H), 4.20(s, 2H). ESI-MS: m/z $[M+H]^+$ calcd for 262.13, found 262.4.

2-Amino-3-benzyl-5-(4-hydroxyphenyl)-pyrazine **(4-14)**: yellow solid; yield 37%; ^{1}HNMR (400 MHz, CD_3OD): δ8.22 (s, 1H), 7.72(d, *J* = 8Hz, 2H), 7.22 (m, 5H), 6.86(d, *J* = 8Hz, 2H), 4.15 (s, 2H). ESI-MS: m/z $[M+H]^+$ calcd for 278.13, found 278.1.

The preparation of 2-amino-3-(5-methylfuryl)-5-(5-methylfuryl)–pyrazine (**4-15**):

2-Amino-3-(5-methylfuryl-2)-5-bromo-pyrazine (**1-2**, 400 mg, 1.58 mmol) was added to a suspension of Pd(dppb)Cl_2 (68 mg, 0.11 mmol) and $(C_6H_5CN)_2PdCl_2$ (43 mg, 0.11 mmol) in toluene (6 mL) and stirred at room temperature under argon atmosphere. 4,4,5,5-tetramethyl-2-(5-methylfuran-2-yl)-1,3,2-dioxaborolane (723 mg, 3.48

mmol) in toluene (4 mL) and then potassium carbonate aqueous solution (2 M, 0.6 mL) was sequentially added to this mixture with stirring. The mixture was heated to reflux at 109°C for 8 h and then allowed to cool to room temperature. The mixture was evaporated under vacuum and redissolved in ethyl acetate. Then it was extracted by ethyl acetate and washed with saturated sodium chloride aqueous solution. After being dried over anhydrous sodium sulfate and concentrated under reduced pressure, the crude product was further purified by chromatography on silica gel (PE/EtOAC10:1) to give yellow solid (100 mg). Yield 25%. ^{1}HNMR (400 MHz, DMSO-d_6): δ8.22 (s,1H), 7.05 (d, J= 4Hz, 1H), 6.77 (d, *J* = 4Hz, 1H), 6.58 (s, 2H), 6.33(d, *J* = 4Hz, 1H), 6.21(d, *J* = 4Hz, 1H), 2.41 (s, 3H), 2.35 (s, 3H). ^{13}CNMR (100 MHz, DMSO-d_6): δ 153.6, 152.1, 150.7, 150.1, 149.6, 136.0, 133.8, 128.7, 112.3, 108.9, 108.5, 107.5, 14.0. ESI-HRMS: m/z $[M+H]^+$ calcd for 256.1086, found 256.1082.

4-16 were prepared with similar method.

2-Amino-3-(5-methylfuryl)-5-(4-hoxyphenyl)–pyrazine (**4-16**): yellow solid; yield 25%. ^{1}HNMR (400 MHz, DMSO-d_6): δ 9.59 (s,1H), 8.41 (s,1H), 7.84 (dd, *J* = 8Hz, 4Hz, 2H), 7.08 (d, *J* = 4Hz, 1H), 6.84 (dd, *J* = 8Hz, 4Hz, 2H), 6.45 (s, 2H), 6.33 (dd, *J* = 4Hz, 4Hz, 1H), 2.43 (s, 3H). ^{13}CNMR (100 MHz, DMSO-d_6): δ 157.9, 153.2, 150.7, 149.4, 140.2, 137.0, 128.3, 128.2, 126.9, 116.0, 111.8, 108.8, 14.1. ESI-HRMS: m/z $[M+H]^+$ calcd for 268.1086, found 268.1083.

The preparation of 2-benzyl-8-benzyl-6-(4-fluorophenyl)imidazo[1,2-a]pyrazin-3(7*H*)-one (**B1**):

The mixture of 2-amino-3-benzyl-5-(4-fluorophenyl)-pyrazine (2-1, 100 mg, 0.35 mmol) and 3-phenyl-1,1-diethoxyacetone (3-1, 160 mg, 0.72 mmol) was dissolved in ethanol (3 mL) under argon atmosphere and allowed to stir at room temperature for 10 min. The con.HCl (0.2 mL) in ethanol (2 mL) was then added to the mixture via syringe over 10 min. The reaction was heated to refluxed at 80°C for 8 h and then allowed to cool to room temperature. The crude was concentrated under vacuum and further purified by chromatography on silica gel (CH_2Cl_2/MeOH 50:1) to give white solid (44 mg). Yield: 30%. Analytical RP HPLC (Phenomenex, C8, 250 x 4.6 mm column): 60% acetonitrile with 0.1% trifluoroacetic acid, 1.0 mL/min at 370 nm, Rt: 5.315 min, 100%. ^{1}HNMR (400 MHz, CD_3OD): δ8.58(s, 1H), 8.02~7.98(m, 2H), 7.40~7.39(m, 2H), 7.34~7.22(m, 10H), 4.55(s, 2H), 4.30(s, 2H). ^{13}CNMR (100 MHz, CD_3OD): δ165.2, 162.7, 136.6, 135.5, 128.9, 128.8, 128.5, 128.4, 128.2, 126.9, 126.8, 115.7, 115.5, 110.1, 37.7, 29.2. ESI-HRMS: m/z $[M+H]^+$ calcd for 410.1669, found 410.1663.

B2, **B3**, **B4**, **B6**, **B7**, **B8**, **B9**, **B10**, **B11**, **B13**, **B14, B15** and **B16** were prepared with similar method.

2-Benzyl-8-benzyl-6-(4-amino-3-fluorophenyl)imidazo[1,2-a]pyrazin-3(7*H*)-one (**B2**): dark yellow solid; yield 42%; Analytical RP HPLC (Phenomenex, C8, 250 x 4.6 mm column): 60% acetonitrile with 0.1% trifluoroacetic acid, 1.0 mL/min at 380 nm, Rt: 5.315 min, 97%. ^{1}HNMR (400 MHz, CD_3OD): δ8.97(s, 1H), 8.14~8.08(m, 2H), 7.69 (t, J=8Hz, 1H), 7.45~7.26(m, 10H), 4.62(s, 2H), 4.38(s, 2H). ^{13}CNMR (100 MHz, CD_3OD): δ157.3, 154.9, 148.2, 139.1, 137.9, 136.5, 135.4, 129.1, 128.3, 128.1, 126.9, 126.8, 125.2, 123.3, 123.3, 119.5, 114.8, 114.6, 111.3, 38.2, 28.8. ESI-HRMS: m/z $[M+H]^+$ calcd for 425.1778, found 425.1777.

2-Benzyl-8-benzyl-6-(4-hydroxymethylphenyl)imidazo[1,2-a]pyrazin-3(7*H*)-one (**B3**): yellow solid; yield 34%; Analytical RP HPLC (Phenomenex, C8, 250 x 4.6 mm column): 55% acetonitrile with 0.1% trifluoroacetic acid, 1.0 mL/min at 380 nm, Rt: 10.261 min, 97%. ^{1}HNMR (400 MHz, CD_3OD): δ7.65(s, 1H), 7.50~7.18(m, 14H), 4.67(s, 2H), 4.44(s, 2H), 4.20(s, 2H). ^{13}CNMR (100 MHz, CD_3OD): δ147.4, 143.5, 139.8, 136.8, 132.5, 129.1, 129.0, 128.7, 128.5, 128.4, 128.3, 127.5, 127.2, 127.1, 126.9, 126.7, 125.3, 125.2, 110.2, 63.2, 37.4, 29.6. ESI-HRMS: m/z [M+ H^+] calcd for 422.1869, found 422.1865.

2-Benzyl-8-benzyl-6-(4-nitrilephenyl)imidazo[1,2-a]pyrazin-3(7*H*)-one (**B4**): brown solid; yield 39%; Analytical RP HPLC (Phenomenex, C8, 250 x 4.6 mm column): 50% acetonitrile with 0.1% trifluoroacetic acid, 1.0 mL/min at 380 nm, Rt: 9.776 min, 96%. ^{1}HNMR (400 MHz, CD_3OD): δ8.88(s, 1H), 8.23(d, J=8Hz, 2H), 7.86(d, J=8Hz, 2H), 7.42~7.25(m, 10H), 4.58(s, 2H), 4.34(s, 2H). ^{13}CNMR (100 MHz, CD_3OD): δ150.7, 148.1, 139.7, 139.0, 137.9, 135.5, 132.5, 132.5, 129.1, 128.6, 128.4, 128.2, 127.2, 126.9, 126.9, 121.9, 118.0, 112.7, 111.6, 38.2, 28.8. ESI-HRMS: m/z $[M+H]^+$ calcd for 417.1715, found 417.1717.

2-Benzyl-8-benzyl-6-(furyl-3)imidazo[1,2-a]pyrazin-3(7*H*)-one (**B6**): yellow solid; yield 43%; Analytical RP HPLC (Phenomenex, C8, 250 x 4.6 mm column): 50% acetonitrile with 0.1% trifluoroacetic acid, 1.0 mL/min at 370 nm, Rt: 4.551 min, 98%. ^{1}HNMR (400 MHz, CD_3OD): δ8.54(s, 1H), 8.19(s, 1H), 7.65(s, 1H), 7.43(d, J=8Hz, 2H), 7.37~7.23(m, 8H), 7.02(d, J=4Hz, 1H), 4.58(s, 2H), 4.34(s, 2H). ^{13}CNMR (100 MHz, CD_3OD): δ147.7, 144.5, 142.3, 136.7, 135.5, 128.9, 128.5, 128.3, 128.2, 126.9, 126.7, 121.7, 109.1, 107.7, 37.4, 29.3. ESI-HRMS: m/z $[M+H]^+$ calcd for 382.1556, found 382.1557.

2-Benzyl-8-benzyl-6-(3-ethyoxycarbonylphenyl)imidazo[1,2-a]pyrazin-3(7*H*)-one (**B7**): dark yellow solid; yield 31%; Analytical RP HPLC (Phenomenex, C8, 250 x 4.6 mm column): 45% acetonitrile with 0.1% trifluoroacetic acid, 1.0 mL/min at 370 nm, Rt: 6.564 min, 98%. ^{1}HNMR (400 MHz, CD_3OD): δ8.78(s, 1H), 8.65(s, 1H), 8.23(d, J=8Hz, 1H), 8.07(d, J=8Hz, 1H), 7.46(d, J=4Hz, 2H), 7.39~7.24(m, 9H), 4.61(s, 2H), 4.42(q, J=8Hz, 2H), 4.40~4.34(m, 2H), 1.43(t, J=8Hz, 3H). ^{13}CNMR (100 MHz, CD_3OD): δ166.2, 150.4, 147.8, 140.1, 138.0, 136.6, 135.5, 134.8, 130.8, 129.0, 128.6, 128.4, 128.2, 126.9, 126.8, 110.6, 61.2, 37.9, 29.1, 13.3. ESI-HRMS: m/z $[M+H]^+$ calcd for 464.1974, found 464.1964.

2-Benzyl-8-benzyl-6-(benzofuranyl-2)imidazo[1,2-a]pyrazin-3(7*H*)-one (**B8**): dark yellow solid; yield 58%; Analytical RP HPLC (Phenomenex, C8, 250 x 4.6 mm column): 45% acetonitrile with 0.1% trifluoroacetic acid, 1.0 mL/min at 380 nm, Rt: 6.564 min, 98%. ^{1}HNMR (400 MHz, CD_3OD): δ8.55(s, 1H), 7.57(t, J=6Hz, 4H), 7.47(d, J=8Hz, 3H), 7.31(t, J=8Hz, 4H), 7.24(m, 3H), 7.13(s, 1H), 4.63(s, 2H), 4.36(s, 2H). ^{13}CNMR (100 MHz, CD_3OD): δ155.0, 151.5, 149.1, 138.0, 137.7, 136.7, 132.8, 129.9, 129.1, 129.0, 128.9, 128.7, 128.4, 127.4, 127.3, 127.1, 126.4, 124.1, 122.3, 120.5, 111.8, 110.3, 106.7, 100.0, 38.1, 29.2. ESI-HRMS: m/z $[M+H]^+$ calcd for 432.1712, found 432.1703.

2-Benzyl-8-benzyl-6-(furyl-2)imidazo[1,2-a]pyrazin-3(7*H*)-one (**B9**): dark yellow solid; yield 30%; Analytical RP HPLC (Phenomenex, C8, 250 x 4.6 mm column): 50% acetonitrile with 0.1% trifluoroacetic acid, 1.0 mL/min at 370 nm, Rt: 4.586 min, 98%. ^{1}HNMR (400 MHz, CD_3OD): δ8.50(s, 1H), 7.69(s, 1H), 7.43~7.27(m, 10H), 7.08(d,

J=4Hz, 1H), 6.60(s, 1H), 4.56(s, 2H), 4.34(s, 2H). ^{13}CNMR (100 MHz, CD_3OD): δ149.2, 148.2, 144.3, 138.0, 136.5, 135.4, 134.0, 128.6, 128.3, 128.1, 121.0, 111.9, 110.5, 107.6, 37.8, 29.0. ESI-HRMS: m/z $[M+H]^+$ calcd for 382.1556, found 382.1545.

2-Benzyl-8-benzyl-6-(4-methoxyphenyl)imidazo[1,2-a]pyrazin-3(7*H*)-one (**B10**): yellow solid; yield 52%; Analytical RP HPLC (Phenomenex, C8, 250 x 4.6 mm column): 55% acetonitrile with 0.1% trifluoroacetic acid, 1.0 mL/min at 370 nm, Rt: 7.431 min, 98%. ^{1}HNMR (400 MHz, CD_3OD): δ7.91(s, 1H), 7.68~7.66(d, J=8Hz, 2H), 7.40~7.38(d, J=8Hz, 2H), 7.32~7.17(m, 8H), 7.04~7.02(d, J=8Hz, 2H), 4.46(s, 2H), 4.21(s, 2H), 3.83(s, 3H). ^{13}CNMR (100 MHz, CD_3OD): δ161.4, 139.6, 138.4, 136.8, 135.6, 128.9, 128.5, 128.4, 128.2, 128.2, 126.9, 126.7, 125.8, 114.2, 109.3, 54.6, 37.4, 29.7. ESI-HRMS: m/z $[M+H]^+$ calcd for 422.1869, found 422.1863.

2-Benzyl-8-benzyl-6-(naphthyl)imidazo[1,2-a]pyrazin-3(7*H*)-one (**B11**): dark yellow solid; yield 12%; Analytical RP HPLC (Phenomenex, C8, 250 x 4.6 mm column): 63% acetonitrile with 0.1% trifluoroacetic acid, 1.0 mL/min at 395 nm, Rt: 4.547 min, 96%. ^{1}HNMR (400 MHz, CD_3OD): δ8.76 (s, 1H), 8.52 (s, 1H), 8.00 (m, 4H), 7.56~7.11 (m, 14H), 4.62 (s, 2H), 4.34 (s, 2H). ^{13}CNMR (100 MHz, CD_3OD): δ150.01, 145.43, 144.34, 139. 60, 135.09, 133.67, 131.44, 129.69, 129.21, 129.05, 128.55, 128.41, 128.38, 128.32, 128.25, 128.17, 127.33, 126.96, 126.82, 126.55, 126.35, 124.93, 124.66, 122.39, 120.53, 38.52, 22.11. ESI-HRMS: m/z $[M+H]^+$ calcd for 442.1919, found 442.1915. ESI-HRMS: m/z $[M+H]^+$ calcd for 442.1919, found 442.1915.

2-Benzyl-8-benzyl-6-(phenyl)imidazo[1,2-a]pyrazin-3(7*H*)-one (**B13, DeepBlueC**): light brown solid; yield 60%; Analytical RP HPLC (Phenomenex, C8, 250 x 4.6 mm column): 50% acetonitrile with 0.1% trifluoroacetic acid, 1.0 mL/min at 380 nm, Rt: 9.824 min, 97%. ^{1}HNMR (400 MHz, CD_3OD): δ7.90(s, 1H), 7.55~7.17(m, 15H), 4.37(s, 2H), 4.11(s, 2H). ^{13}CNMR (100 MHz, CD_3OD): δ147.9, 139.6, 138.0, 137.8, 136.8, 136.7, 134.5, 130.1, 129.8, 129.5, 129.1, 129.0, 128.9, 127.4, 127.2, 127.0, 126.4, 111.2, 38.0, 29.5. ESI-HRMS: m/z $[M+H]^+$ calcd for 392.1763, found 392.1755.

2-Benzyl-8-benzyl-6-(4-hydroxyphenyl)imidazo[1,2-a]pyrazin-3(7*H*)-one **(B14, CTZ h)**: yellow solid; yeild 20%; Analytical RP HPLC (Phenomenex, C8, 250 x 4.6 mm column): 35% acetonitrile with 0.1% trifluoroacetic acid, 1.0 mL/min at 325 nm, Rt: 11.892 min, 99%. ^{1}HNMR (400 MHz, CD_3OD): δ 8.38 (s, 1 H), 7.71 (d, *J*=8Hz, 2H), 7.46 (m, 2H), 7.35~7.25 (m, 8H), 6.86 (d, *J* = 8Hz, 2H), 4.57 (s, 2H), 4.34 (s, 2H). ESI-HRMS: m/z $[M+H]^+$ calcd for 408.17129, found 408.1705.

2-Benzyl-6-(5-methylfuryl)-8-(5-methylfuryl)imidazo[1,2-a]pyrazin-3(7*H*)-one (**B15**): red solid; yield 12%. Analytical RP HPLC (Phenomenex, C8, 250 x 4.6 mm column): 50% acetonitrile with 0.1% trifluoroacetic acid, 1.0 mL/min at 420 nm, Rt: 10.589 min, 100%. ^{1}HNMR (400 MHz, CD_3OD): δ8.34 (s, 1H), 7.59 (d, *J* = 4Hz, 1H), 7.37~7.31 (m, 5H), 7.09 (d, *J* = 4Hz, 1H), 6.47 (dd, *J* = 4, 0.8Hz, 1H), 6.26 (dd, *J* = 4, 0.8Hz, 1H), 4.40 (s, 2H), 2.56 (s, 3H), 2.45 (s, 3H). ^{13}CNMR (100 MHz, CD_3OD): δ156.6, 153.3, 149.3, 147.3, 139.3, 137.5, 137.2, 132.1, 129.0, 128.8, 126.7, 118.1, 110.4, 109.8, 108.9, 106.8, 30.8, 14.3, 14.0. ESI-HRMS: m/z $[M+H]^+$ calcd for 386.1505, found 386.1504.

2-Benzyl-6-(4-hydroxyphenyl)-8-(5-methylfuryl)imidazo[1,2-a]pyrazin-3(7*H*)-one (**B16**): red solid; yield 26%.

Analytical RP HPLC (Phenomenex, C8, 250 x 4.6 mm column): 45% acetonitrile with 0.1% trifluoroacetic acid, 1.0 mL/min at 450 nm, Rt: 7.407 min, 95%. ^{1}HNMR (400 MHz, CD_3OD): δ10.99 (s, 1H), 9.64 (s, 1H), 8.27 (s, 1H), 7.93 (d, J = 8Hz, 2H), 7.87 (d, J = 2.8Hz, 1H), 7.34~7.16 (m, 5H), 6.88 (d, J = 8Hz, 2H), 6.38 (d, J = 2.4Hz, 1H), 4.11 (s, 2H), 2.45 (s, 3H). ^{13}CNMR (100 MHz, CD_3OD): δ158.2, 155.2, 148.2, 140.6, 138.0, 137.0, 129.0, 128.7, 128.3, 128.1, 127.7, 126.4, 118.3, 115.9, 109.3, 107.7, 32.6, 14.2. ESI-HRMS: m/z $[M+H]^+$ calcd for 398.1505, found 386.1498.

Organic synthesis of T-series

The preparation of 2-amino-3-benzyl- 5-((trimethylsilyl)ethynyl)-pyrazine (**5**):

The suspension of $PdCl_2(PPh_3)_2$ (27 mg, 0.038 mmol), CuI (29 mg, 0.151 mmol) and PPh_3 (20 mg, 0.076 mmol) in 2 mL toluene was added to the solution of 3-benzyl-5-bromo-2-amino-pyrazine (200mg, 0.76mmol) in 3 mL toluene under argon atmosphere. Then trimethylsilylacetylene (223 mg, 0.27 mmol) and triethylamine (0.8 mL) were sequentially added. The mixture was stirred at 40°C for 9 h. Then the mixture was filtered and the filtrate was collected and evaporated under vacuum. The collection was extracted by ethyl acetate and washed with saturated sodium chloride solution. After dried over anhydrous sodium sulfate and concentrated under reduced pressure, the crude product was further purified by chromatography on silica gel (PE/EtOAC 10:1) to give light yellow solid (129 mg). Yield: 60%.^{1}HNMR (400 MHz, DMSO-d6): δ 7.98 (s, 1 H), 7.29~7.20 (m, 5H), 6.79 (s, 2 H), 3.99 (s, 2H). ESI-MS: m/z $[M+H]^+$ calcd for 282.14, found 282.5.

The preparation of 2-amino-3-benzyl- 5-ethynyl-pyrazine (**6**):

Potassium carbonate (40 mg, 0.284 mmol) was added to the solution of 2-amino-3-benzyl-5-((trimethylsilyl)ethynyl)-pyrazine (100 mg, 0.356 mmol) in methanol and the mixture was stirred at room temperature for 2 h. Then the reagent was evaporated under vacuum and the crude was redissolved in ethyl acetate. The organic layer was washed with saturated sodium chloride solution after extraction with ethyl acetate. After dried over anhydrous sodium sulfate and concentrated under reduced pressure, the crude product was further purified by chromatography on silica gel (PE/EtOAC 20:3) to give light yellow solid (50 mg). Yield: 67%. ^{1}HNMR (400 MHz, DMSO-d6): δ 7.98 (s, 1 H), 7.30~7.20 (m, 5H), 6.76 (s, 2 H), 4.15 (s, 2H), 3.99(s, 2H). ESI-MS: m/z [M+ +] calcd for 210.10, found 210.3.

The preparation of 2-azidoethanol (**7-1**):

2-Bromoethanol (1.76 g, 14.08 mmol) was dissolved in water (3mL) and then sodium azide (1.83 g, 28.17 mmol) was added to this mixture with stirring. The mixture was stirred at room temperature for 20 h. The mixture was extracted with diethyl ether and the organic layer was washed with saturated sodium chloride solution. After dried over anhydrous sodium sulfate and concentrated under reduced pressure, the crude product was applied to the next reaction without further purification.

7-2 and **7-3** were prepared with similar method.

The preparation of 2-amino-3-benzyl- 5- [(1-hydroxyethyl)-1, 2, 3- triazol-4]-pyrazine (**8-1**):

Copper sulfate pentahydrate (2 mg, 7.15 μmol) and sodium ascorbate (4 mg, 1.45 μmol) in water (1.5 mL) were sequentially added to the mixture of 2-amino-3-benzyl-5-ethynyl-pyrazine (30 mg, 0.143 mmol) and 2-azidoethanol (16 mg, 0.186 mmol) in THF (1.5 mL) under argon. Then triethylamine (2 μL) was added and the mixture was stirred at room temperature for 8 h. Then the mixture was extracted with ethyl acetate and the organic layer was washed with saturated sodium chloride solution. After dried over anhydrous sodium sulfate and concentrated under reduced pressure, the crude product was further purified by chromatography on silica gel (PE/EtOAC 20:3) to give light yellow solid (40 mg). Yield: 95%. ^{1}HNMR (400 MHz, $CDCl_3$): δ 8.70 (s, 1H), 8.10(s, 1H), 7.34~7.28 (m, 2H), 7.26~7.22(m, 3H), 4.55(t, J=4Hz, 2H), 4.49(s, 2H), 4.13(m, 4H). ESI-MS: m/z $[M+H]^+$ calcd for 297.1464, found 297.1456.

8-2 and **8-3** were prepared with similar method.

2-Amino-3-benzyl- 5- [(1-hydroxypropyl)-1, 2, 3- triazol-4]-pyrazine (**8-2**): light yellow solid; yield 83%; ^{1}HNMR (400 MHz, $CDCl_3$): δ 8.76 (s, 1H), 8.04(s, 1H), 7.33~7.28 (m, 2H), 7.26~7.22(m, 3H), 4.59(t, *J*=6Hz, 2H), 4.48(s, 2H), 4.14(s, 2H), 3.70(m, *J*=6Hz, 2H), 2.19(m, 2H). ESI-MS: m/z $[M+H]^+$ calcd for 311.16, found 311.3.

2-Amino-3-benzyl- 5- [(1-fluoroethyl)-1, 2, 3- triazol-4]-pyrazine (**8-3**): light yellow solid; yield 79%; ^{1}HNMR (400 MHz, DMSO-d6): δ 8.45 (s, 1H), 8.39(s, 1H), 7.33~7.19 (m, 5H), 6.46(s, 2H), 4.93(t, *J*=4Hz, 1H), 4.80(m, 2H), 4.73(m, 2H), 4.07(s, 2H). ESI-MS: m/z $[M+H]^+$ calcd for 299.14, found 299.5。

The preparation of 2-benzyl-8-benzyl-6-[(1-hydroxyethyl)-1, 2, 3-triazol-4]imidazo[1,2-a]pyrazin-3(7*H*)-one (**T1**):

The mixture of 2-amino-3-benzyl-5-[(1-hydroxyethyl)-1, 2, 3-triazol-4]-pyrazine (8-1, 60 mg, 0.202 mmol) and 3-phenyl-1,1-diethoxyacetone (3-1, 90 mg, 0.404 mmol) was dissolved in ethanol (3 mL) under argon atmosphere and allowed to stir at room temperature for 10 min. The con.HCl (0.2 mL) in ethanol (1 mL) was then added to the mixture via syringe over 10 min. The reaction was heated to refluxed at 80°C for 8 h and then allowed to cool to room temperature. The crude was concentrated under vacuum and further purified by chromatography on silica gel (CH_2Cl_2/MeOH 50:1) to give yellow solid (60 mg). Yield: 69%. Analytical RP HPLC (Phenomenex, C8, 250 x 4.6 mm column): 45% acetonitrile with 0.1% trifluoroacetic acid, 1.0 mL/min at 370 nm, Rt: 3.923 min, 98%. ^{1}HNMR (400 MHz, CD_3OD): δ 8.35 (s, 1H), 8.02(s, 1H), 7.24~7.05 (m, 10H), 4.44(t, *J*=6Hz, 2H), 4.32(s, 2H), 4.04(s, 2H), 3.86(t, *J*=6Hz, 2H). ^{13}CNMR (100 MHz, CD_3OD): δ148.40, 143.06, 138.07, 136.52, 135.47, 133.85, 128.86, 128.54, 128.37, 128.17, 126.88, 126.80, 124.56, 109.73, 60.10, 52.93, 37.81, 29.08. ESI-HRMS: m/z $[M+H]^+$ calcd for 427.1882, found 427.1876.

T2 and **T3** were prepared with similar method.

2-Benzyl-8-benzyl-6- [(1-hydroxypropyl)-1, 2, 3- triazol-4]imidazo[1,2-a]pyrazin-3(7*H*)-one (**T2**): yellow solid; yield 23%. Analytical RP HPLC (Phenomenex, C8, 250 x 4.6 mm column): 50% acetonitrile with 0.1% trifluoroacetic acid, 1.0 mL/min at 370 nm, Rt: 3.888 min, 100%. ^{1}HNMR (400 MHz, CD_3OD): δ 8.44 (s, 1H),

8.15(s, 1H), 7.35~7.12 (m, 10H), 4.57(t, *J*=8Hz, 2H), 4.44(s, 2H), 4.14(s, 2H), 3.58(t, *J*=8Hz, 2H), 2.13(m, 2H). ^{13}CNMR (100 MHz, CD_3OD): δ148.42, 143.09, 136.51, 135.48, 133.72, 128.84, 128.55, 128.39, 128.16, 126.88, 126.80, 124.19, 109.90, 65.52, 57.92, 37.84, 32.49, 29.07. ESI-HRMS: m/z $[M+H]^+$ calcd for 441.2039, found 441.2034.

2-Benzyl-8-benzyl-6- [(1-fluoroethyl)-1, 2, 3- triazol-4]imidazo[1,2-a]pyrazin-3(7*H*)-one (**T3**): yellow solid; yield 25%. Analytical RP HPLC (Phenomenex, C8, 250 x 4.6 mm column): 60% acetonitrile with 0.1% trifluoroacetic acid, 1.0 mL/min at 370 nm, Rt: 3.035 min, 95%. ^{1}HNMR (400 MHz, CD_3OD): δ 8.47 (s, 1H), 8.26(s, 1H), 7.41~7.20 (m, 10H), 4.92(m, 1H), 4.85(m, 3H), 4.46(s, 2H), 4.21(s, 2H). ^{13}CNMR (100 MHz, CD_3OD): δ148.43, 143.66, 138.03, 136.52, 135.46, 134.00, 128.86, 128.55, 128.38, 128.17, 126.88, 126.81, 124.36, 109.68, 82.24, 80.54, 37.85, 29.03. ESI-HRMS: m/z $[M+H]^+$ calcd for 429.1839, found 429.1833.

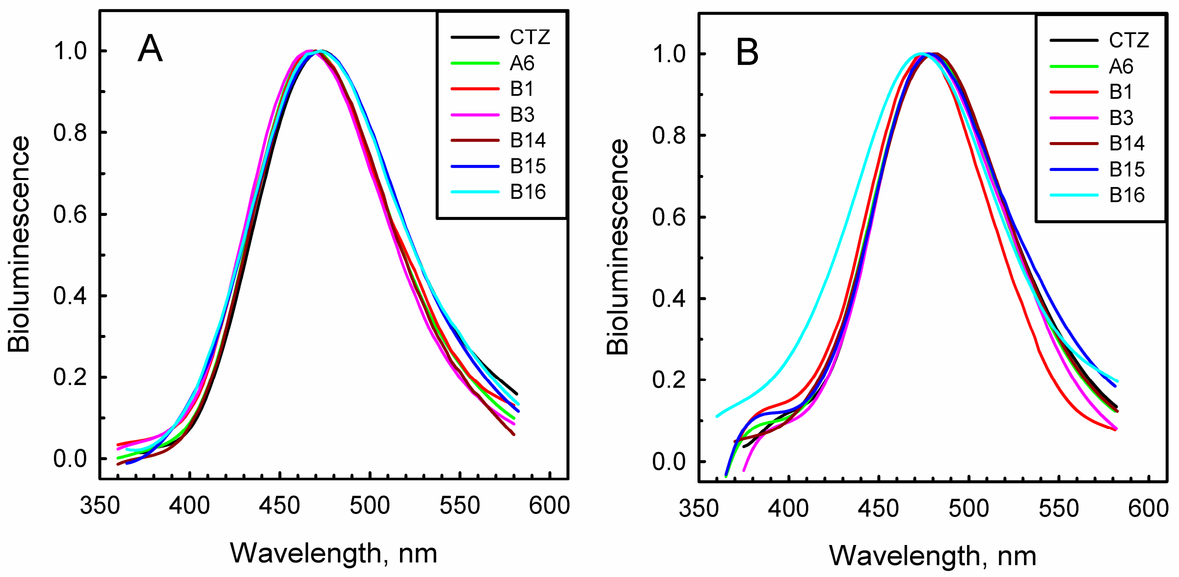

Figure S1. Bioluminescence spectra of aequorins (A) and obelins (B) activated with native coelenterazine and some coelenterazine analogues.

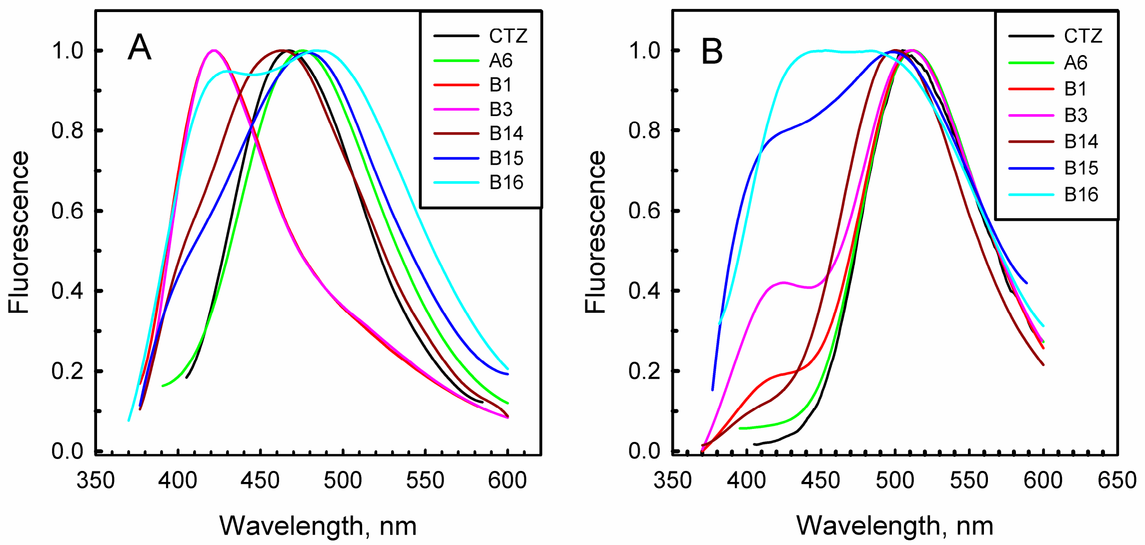

Figure S2. Fluorescence spectra of Ca^{2+}-discharged aequorins (A) and obelins (B) activated with native coelenterazine and some coelenterazine analogues.

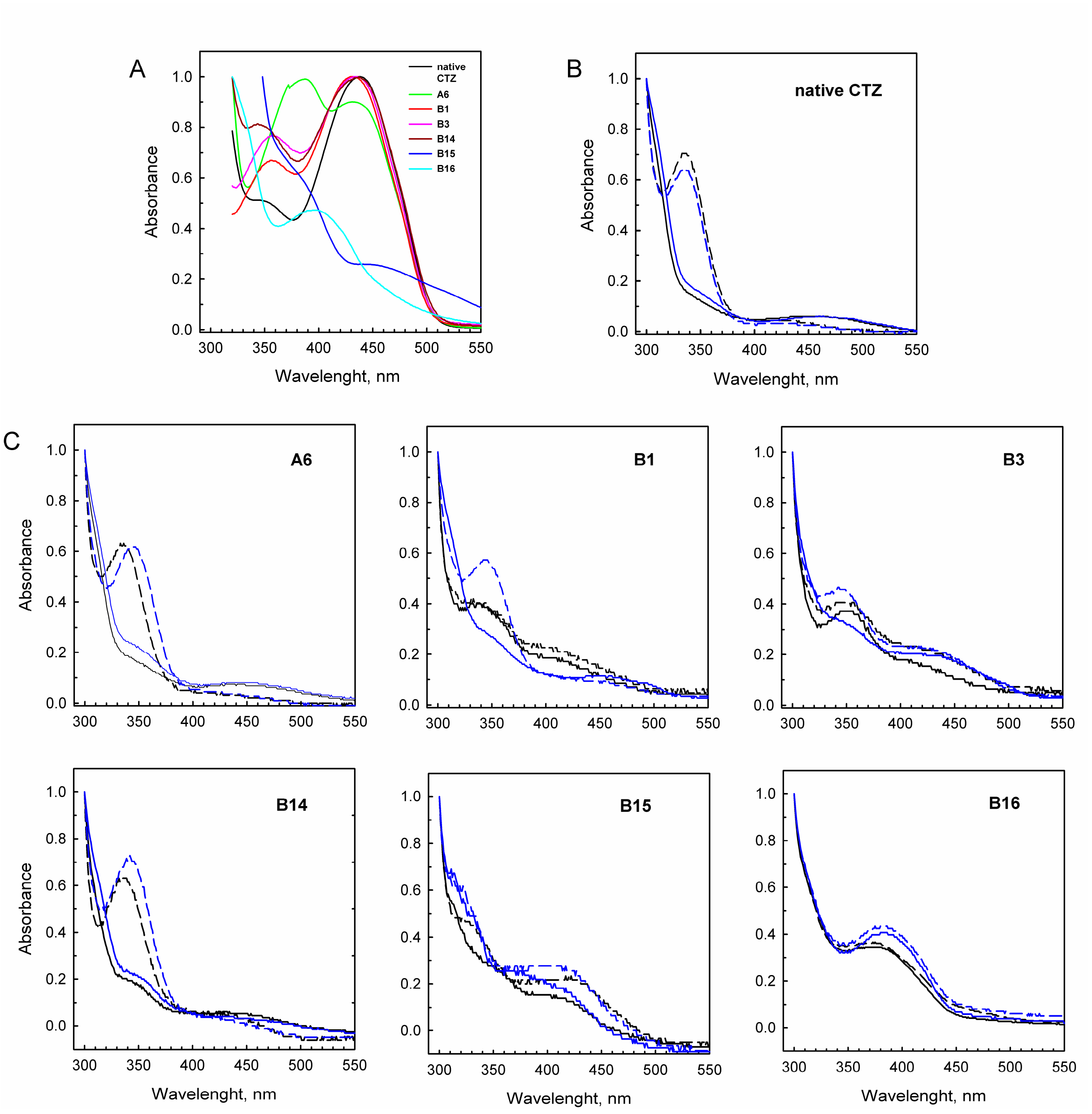

Figure S3. Absorption spectra of native coelenterazine and some coelenterazine analogues in ethanol (A), photoproteins activated by native coelenterazine (B) and some coelenterazine analogues (C). Active aequorin and obelin are shown with solid black and blue lines, respectively; Ca^{2+}-discharged aequorin and obelin are shown with dashed black and blue lines, respectively.
